# Detection of a Target Nucleic Acid by Ligation‐Assisted Fluorescence Enhancement of a Peptide Nucleic Acid (PNA) Twin Probe via Disulfide Binding

**DOI:** 10.1002/bip.70105

**Published:** 2026-05-15

**Authors:** Yutaka Ouchi, Koki Ishii, Yumiko Sato, Yoshitane Imai, Hideo Matsui, Takashi Ohtsuki, Yoshiyuki Hakata, Hajime Shigeto, Shohei Yamamura, Mizuki Kitamatsu

**Affiliations:** ^1^ Department of Applied Chemistry Kindai University Higashiosaka Japan; ^2^ Department of Interdisciplinary Science and Engineering in Health Systems Okayama University Okayama Japan; ^3^ Department of Arts and Sciences Faculty of Medicine, Kindai University Sakai Japan; ^4^ Health and Medical Research Institute, National Institute of Advanced Industrial Science and Technology (AIST) Takamatsu Japan

**Keywords:** cell‐penetrating peptide, disulfide crosslinking, fluorescent probe, peptide nucleic acid, pyrene

## Abstract

The development of methods for detecting specific nucleic acids is important for early diagnosis and treatment of diseases at the genetic level. We have developed a pair of pyrene (Pyr)‐modified peptide nucleic acids (PNAs), PNA twin probe, as a tool for such detection. In this study, we prepared Pyr‐PNAs containing chloroacetyl (‐COCH_2_Cl) or thiol (‐SH) groups at the termini by solid‐phase peptide synthesis. By analyzing various candidates, we clarified that a pair of Pyr‐PNAs, each containing an SH group, formed a disulfide bond through the hybrid formation of two PNAs with complementary DNA, resulting in excimer emission at 455 nm. Furthermore, we demonstrated that these Pyr‐PNAs provide fluorescent detection of intracellular target RNAs through enhanced excimer emission via the ligation. This work should aid future studies aimed at the specific fluorescent detection of RNA in living cells.

## Graphical abstract

Target‐induced disulfide ligation of PNA twin probes promotes pyrene excimer formation for nucleic acid detection.

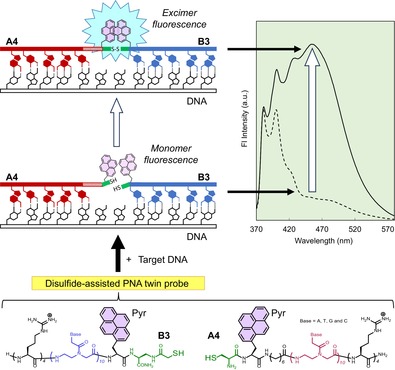

## 1 Introduction

Peptide nucleic acids (PNAs) are artificial nucleic acid analogues that have excellent potential as probes for detecting nucleic acids (DNA and RNA) with specific sequences. This is because PNAs are resistant to nucleases and proteases and form duplexes with DNA or RNA that are more thermally stable than corresponding DNA/DNA and DNA/RNA hybrids. PNAs also exhibit higher sequence specificity than natural DNA probes. It has been reported that the presence of a single mismatch in the target DNA decreases the melting temperature (*T*_m_) by 4°C–16°C for DNA/DNA duplexes and by 8°C–21°C for PNA/DNA duplexes [1, 2]. Based on these properties, PNA‐based electrochemical biosensors [3, 4] and fluorescent probes [5] have been developed, and numerous highly accurate and reliable detection methods have been reported.

Among these, PNA‐based electrochemical biosensors have attracted considerable attention as promising probe systems because their charge‐neutral backbone suppresses nonspecific electrostatic interactions, thereby reducing background signals [6, 7, 8, 9, 10, 11, 12, 13, 14, 15, 16]. These platforms enable highly sensitive and selective nucleic acid detection through electrochemical readout strategies that exploit signal changes associated with hybridization. In recent years, their performance has been significantly improved through integration with nanostructured materials [6, 7, 8], signal amplification chemistries [9, 10, 11, 12], polymer imprinting techniques [13], and DNA origami structures [14], leading to ultrahigh sensitivity for the detection of nucleic acids including miRNA and viral DNA. Further applications have been demonstrated in microbial detection [8, 9] and in performance enhancement via lateral spatial control of polymer brushes on two‐dimensional interfaces [15]. In addition, progress has been made in in vivo‐to‐in vitro signal conversion technologies for disease monitoring using urine biopsy samples [16]. Overall, these studies suggest that PNA‐based hybrid biosensing platforms are versatile and powerful tools for highly sensitive and selective electrochemical nucleic acid detection.

PNA‐based fluorescent probes have also been extensively developed as versatile scaffolds for nucleic acid sensing, owing to their high binding affinity. These probes have been further extended into functional systems capable of converting biological events into detectable optical signals [17, 18, 19, 20, 21, 22, 23, 24, 25, 26]. In recent years, such platforms have enabled activity‐to‐signal conversion, in which enzymatic activities such as proteases are translated into nucleic acid or fluorescence outputs [17, 18]. FIT (forced intercalation technique)‐type and triplex‐forming PNA probes have been developed for the recognition of structured RNAs, including viral RNA and double‐stranded RNA [19, 20]. Importantly, recent advances include signal enhancement strategies based on surrogate base design and photonic engineering. These include red‐shifted bisquinoline fluorophores [21], chemically modified FIT‐PNAs for improved cellular imaging performance [22], and high‐brightness systems utilizing FRET and light‐harvesting mechanisms [23]. These approaches have also been applied to diagnostic applications such as drug resistance detection [24]. Furthermore, PNA technology has been extended to nucleic acid structural analysis and microbial identification, including studies on G‐quadruplex structures [25] and rapid bacterial detection using optimized PNA‐FISH (fluorescence in situ hybridization) systems [26].

Motivated by these fluorescent PNA‐based sensing strategies, we focus on nucleic acid detection methods that combine fluorescence readout with the structural advantages of PNA, as such systems are relatively easy to handle and allow direct visual detection. In our previous work [27, 28], we demonstrated that two Pyr‐labeled PNAs (PNA twin probe) exhibit a fluorescence response that changes from monomer emission to excimer emission upon hybridization with a target DNA, enabling its detection. We also showed that the distance and relative orientation of Pyr moieties in the DNA‐bound complex significantly affect excimer emission. However, the observed excimer‐to‐monomer emission ratio was approximately 1.2, suggesting that further improvement is still possible.

Although the previous system was promising, the two Pyr moieties are likely to retain a certain degree of conformational flexibility, which may prevent the formation of a fully stabilized excimer state. In this study, we therefore designed a strategy to covalently link the two Pyr‐bearing PNAs upon hybridization on the DNA template. We hypothesized that restricting the relative motion of the two Pyr moieties by covalent linkage would lead to more stable excimer formation and enhanced fluorescence emission. To our knowledge, only one related approach has been reported by Seitz et al., in which fluorescence detection of nucleic acids was achieved via native chemical ligation using a combination of fluorescein and tetramethylrhodamine [29]. In the present study, we investigate suitable reactive groups and optimal inter‐PNA distances for ligation using a PNA twin probe system.

## 2 Material and Methods

### 2.1 Materials

9‐Fluorenylmethyloxycarbonyl group (Fmoc)‐protected amino acids, Fmoc‐derivatized super acid‐labile poly(ethylene)glycol (Fmoc‐NH‐SAL‐PEG) resin, Fmoc‐Ala(Pyn)‐OH (a Pyr derivative), Fmoc‐protected alkyl linkers [Fmoc‐NH‐(CH_2_)_6_‐COOH], Fmoc‐Arg(Pbf)‐OH, Fmoc‐Cys(Trt)‐OH, Fmoc‐Lys(ivDde)‐OH, piperidine, *O*‐(1H‐benzotriazol‐1‐yl)‐*N*,*N*,*N′*,*N′*‐tetramethyluronium hexafluorophosphate (HBTU), *N*‐methylmorpholine (NMM), trifluoroacetic acid (TFA), ethanedithiol (EDT), and triisopropylsilane (TIPS) were purchased from Watanabe Chemicals (Hiroshima, Japan). Fmoc‐protected PNA monomers [Fmoc‐A(Bhoc)‐OH, Fmoc‐T‐OH, Fmoc‐G(Bhoc)‐OH, and Fmoc‐C(Bhoc)‐OH] were purchased from Panagene (Daejeon, South Korea). Fmoc‐Dpr(ivDde)‐OH was purchased from Novabiochem (Tokyo, Japan). Fmoc‐Dab(ivDde)‐OH, Fmoc‐Orn(ivDde)‐OH, and 2‐(tritylthio)acetic acid were purchased from BLD Pharm (Shanghai, China). *N*,*N′*‐Dimethylformamide (DMF), *N*‐methyl‐2‐pyrrolidinone (NMP), diethyl ether, acetonitrile, hydrazine monohydrate, dichloromethane (DCM), and HEPES were purchased from FUJIFILM Wako Pure Chemical Corporation (Osaka, Japan). Phosphate‐buffered saline (PBS; 100 mM, pH 7.0) and RPMI 1640 were purchased from Nacalai Tesque (Kyoto, Japan). DNA oligomers and 100 U/mL penicillin–streptomycin were purchased from Thermo Fisher Scientific (Waltham, MA, USA). 10% fetal bovine serum (FBS) was purchased from Sigma‐Aldrich (St. Louis, MO, USA). FITC‐Annexin V was purchased from Biolegend (San Diego, CA, USA). *N*‐(Chloroacetoxy)succinimide (Cl‐CH_2_‐OSu), prepared as previously reported [30], was used in this study.

### 2.2 Synthesis of Peptides

All Pyr‐PNA probes (**A1**–**A4** and **B1**–**B7**; Figure 2) were prepared on Fmoc‐NH‐SAL‐PEG resin with a loading of 7.2 μmol Fmoc/g on its surface using conventional Fmoc‐based solid‐phase peptide synthesis. Deprotection and coupling processes were carried out at room temperature. No capping step was performed. The deprotection of Fmoc was carried out using 20% piperidine in DMF for 7 min. For each coupling process, 4 equiv. Fmoc‐protected PNA monomers, Fmoc‐protected alkyl linkers, Fmoc‐Cys(Trt)‐OH, Fmoc‐Arg(Pbf)‐OH, Fmoc‐Dpr(ivDde)‐OH, Fmoc‐Dab(ivDde)‐OH, Fmoc‐Orn(ivDde)‐OH, Fmoc‐Lys(ivDde)‐OH, or Fmoc‐Ala(Pyn)‐OH, 3.6 equiv. HBTU, and 11.5 equiv. NMM were dissolved in DMF or a DMF/NMP mixture and added to the resin. The reaction was allowed to proceed for 40 min. Coupling and deprotection steps were continued until the desired peptide was elongated. **A1**, **A4**, and **B1** possessed free N‐terminal amino groups, and the amino termini of **B2**–**B7** were acetylated. The amino termini of **A2** and **A3** were coupled with Cl‐CH_2_‐OSu and 2‐(tritylthio) acetic acid, respectively. To further deprotect the ivDde group on the side‐chain amino groups of **B2**–**B7**, the group was treated with 5% hydrazine in DMF for 10 min three times, and then **B2** was coupled with Cl‐CH_2_‐OSu, **B4** with Cys, and **B3** and **B5**–**B7** with 2‐(tritylthio) acetic acid. After the final step, the resin was washed with DCM and then the peptides on the resin were globally deprotected and cleaved from the resin by treatment with 95:2.5:2.5 (v/v) TFA/TIPS/water for 90 min at room temperature. If the peptide contained a thiol group, it was treated with 92.5:2.5:2.5:2.5 (v/v) TFA/EDT/TIPS/water for 90 min at room temperature. Crude peptides were precipitated in diethyl ether and washed with diethyl ether until neutral pH was reached. Peptides were then dried by air and dissolved in 0.1% TFA in water. Peptides were purified using reverse‐phase high‐pressure liquid chromatography (HPLC) on a C18 preparative column (Cadenza 5CD‐C18; Imtakt, Kyoto, Japan) with a linear gradient of eluent A (0.1% TFA in water) and eluent B (acetonitrile). The detection was carried out at 340 nm and the flow rate was 10.0 mL/min. Final product identification was performed using matrix‐assisted laser desorption/ionization‐time‐of‐flight (MALDI‐TOF) mass spectrometry (Shimadzu MALDI‐7090) (Figures S1, S2) and HPLC on a C18 analytical column (Cadenza CD‐C18; Imtakt) (Figures S3, S4). We also identified the final compounds from UV–vis spectroscopy (using a JASCO V‐560 UV–vis spectrometer).

### 2.3 Fluorescence Spectroscopy

Fluorescence measurements were performed using a JASCO FP‐8200 fluorescence spectrometer and a 1 cm quartz cell. Fluorescence spectra of PNA twin probe (**A1**–**A4** and **B1**–**B7**) with or without DNA (**D1**–**D13**) were measured at 25°C in aqueous buffer (10 mM PBS, pH 7.0). The final concentration of Pyr‐PNAs and DNA were both 1.0 μM. The excitation wavelength was 350 nm, and the emission wavelengths range was from 370 to 580 nm. The fluorescence spectra were normalized with the fluorescence intensity at 380 nm derived from the Pyr monomer set as 1.0. The mixture solutions were measured after incubation for the predetermined time. The Pyr‐PNAs were stored as dry powders and dissolved in distilled water immediately prior to use, followed by dilution into the appropriate buffer solution. The concentrations were determined based on the UV absorbance at 260 nm of the probes. All experiments were performed using buffer solutions prepared under atmospheric conditions, without any additional degassing or special pretreatment prior to measurement.

### 2.4 Fluorescence Microscopy Images

Human lung cancer cell lines, PC9 cells (Japan Bioresource Collection Cell Bank, Osaka, Japan) and A549 cells (ATCC, Manassas, VA, USA), were cultured in RPMI 1640 supplemented with 10% FBS, 100 U/mL penicillin–streptomycin, and 25 mM HEPES. Both cells were maintained in a 5% CO_2_ incubator at 37°C. Cells (0.5 × 10^4^) were seeded on a 24 well plate (Greiner Bio‐One GmbH, Kremsmünster, Austria) 1 day prior to PNA treatment. The next day, PNAs were diluted in culture medium and combined to prepare 10 μM respective PNA pairs. The cell culture medium was removed, and 300 μL of 10 μM **A1**/**B1** or **A4**/**B3** PNA pair was added to the cells. After 3 h or 24 h incubation, image acquisition and quantitative analysis were performed using CellVoyager CQ1 equipped with the CellPathfinder built‐in analysis software (Yokogawa Electric Corporation, Tokyo, Japan). The PNA twin probe–derived fluorescence images were acquired using a 405 nm excitation laser, and the emission was collected in the 417–477 nm range. Fluorescent intensities were quantified by measuring the signal for each individual cell, and the mean fluorescence intensity per cell was used for subsequent analyses. For the cytotoxic assay, the **A4**/**B3** PNA pair stimulated cells were treated with FITC‐Annexin V for 30 min and fluorescence images were acquired using a 488 nm excitation laser, and the emission was collected in the 500–550 nm range.

## 3 Results and Discussion

### 3.1 Design

**FIGURE 1:**
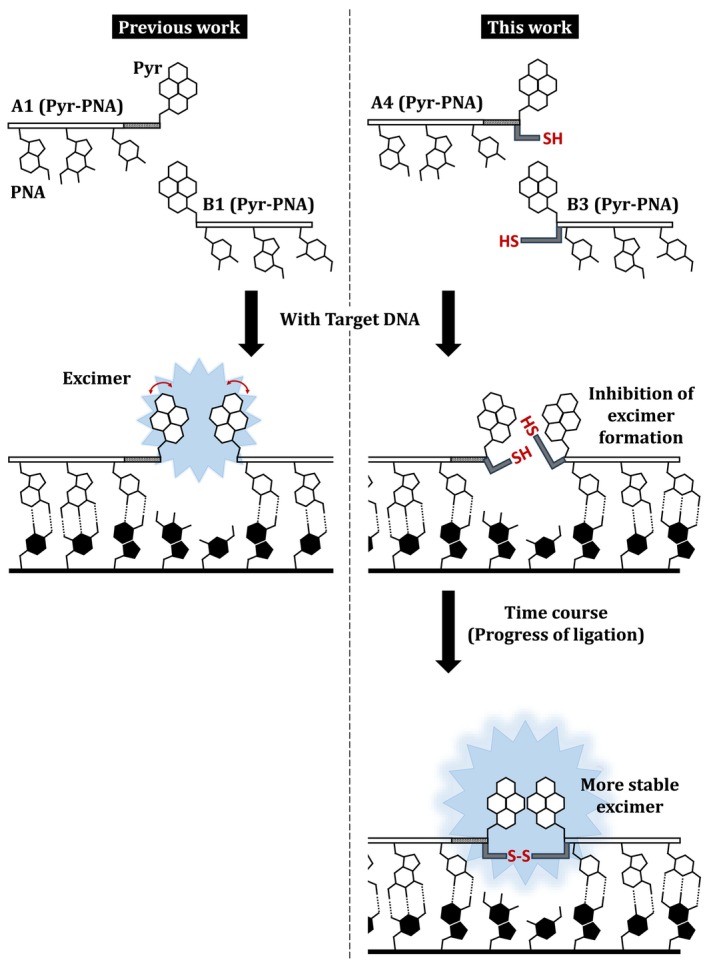
Schematic illustration of PNA twin probe for fluorescence detection of target nucleic acids. The PNA twin probe used in our previous work (left) and the reactive PNA twin probe used in this work (right).

A schematic illustration of this study is shown in Figure 1. In the PNA twin probe used in our previous study, the target DNA and two Pyr‐PNAs form a hybrid, and the DNA acts as a template to orient the two Pyr moieties so that they face each other, thereby promoting excimer formation and fluorescence emission. In contrast, the Pyr‐PNAs used in this study are characterized by the introduction of reactive groups adjacent to the Pyr moieties. When the target DNA and the two Pyr‐PNAs form a hybrid, their reactive groups, like the two Pyr units, are also positioned facing each other.

Prior to the reaction, these reactive groups introduce steric hindrance and inhibit excimer formation between the Pyr moieties. However, over time, the two PNA strands become covalently linked via a reaction between the reactive groups. As a result, after the reaction, the aforementioned steric hindrance is removed, and the reactive groups instead contribute to facilitating excimer formation. Thus, the reactive PNA twin probe in this study is expected to exhibit time‐dependent and stronger fluorescence emission compared with the PNA twin probe reported in our previous work.

**FIGURE 2:**
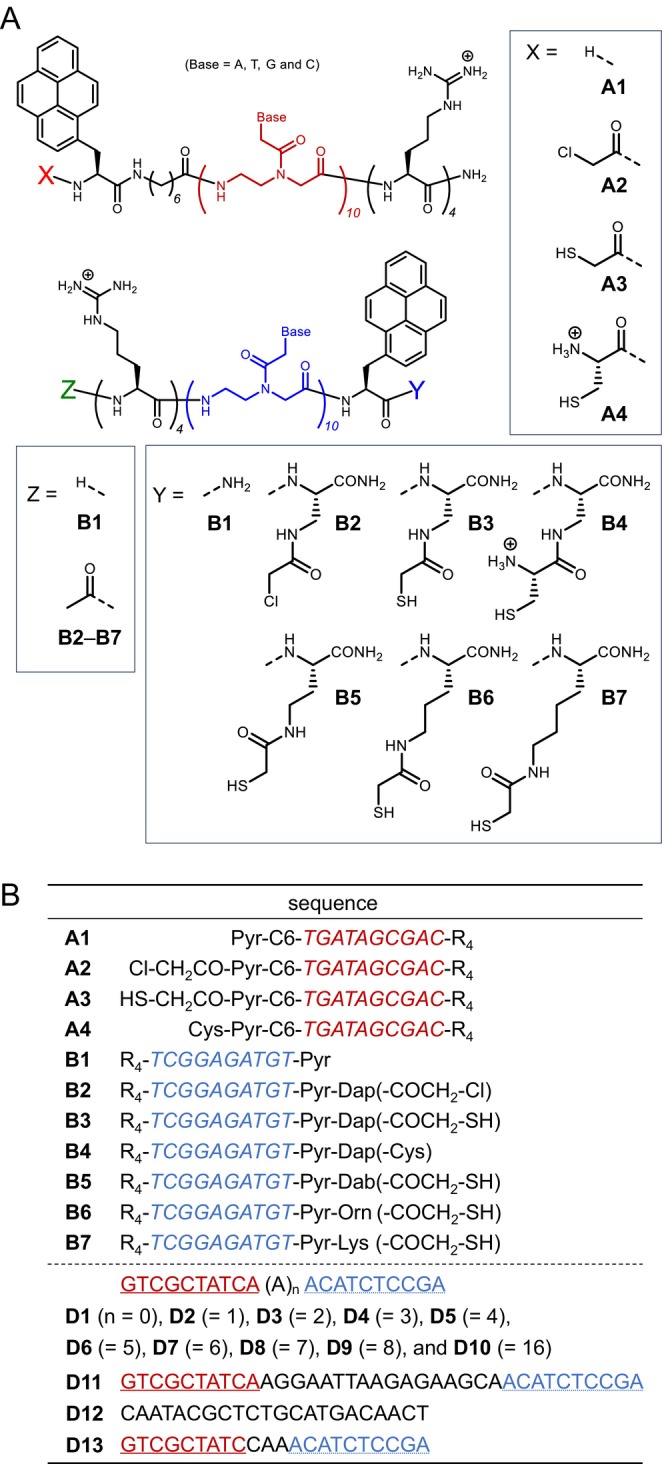
(A) Chemical structures of Pyr‐PNAs (PNA twin probe: **A1**–**A4** and **B1**–**B7**). (B) Sequences of the Pyr‐PNAs and DNA **D1**–**D13**. C6 represents an alkyl linker [‐NH‐(CH_2_)_6_‐CO‐]. Dap, Dab, and Orn represent diaminopropanoic acid, diaminobutanoic acid, and ornithine backbone, respectively. PNA sequences are shown in italics. Underlines and dotted underlines in DNA represent complementary sequences to the **A** series and **B** series, respectively.

In pursuit of the above approach, we designed and synthesized a series of 11 Pyr‐PNAs, **A1**–**A4** and **B1**–**B7** (Figure 2). **A1**–**A4** consist of a 10‐mer PNA (base sequence: TGATAGCGAC) whose N‐terminus is modified with a pyrenylalanine via a C6 linker [‐NH‐(CH_2_)_6_‐CO‐] and whose C‐terminus is conjugated with tetraarginine (R_4_). **B1**–**B7** consist of a 10‐mer PNA (base sequence: TCGGAGATGT) whose C‐terminus is modified with a pyrenylalanine and whose N‐terminus is conjugated with R_4_.

The R_4_ is expected to play a role as a cell‐penetrating peptide (CPP) [31, 32], as well as to improve the water solubility of the PNA probe. Although the R_4_ seems short compared with typical oligoarginine‐based CPPs, we previously reported that even triarginine and tetraarginine can be introduced into cells [33, 34]. **A1** and **B1** have the same structures as those used in our previous study [27].

In addition to the **A1** structure, the N‐termini of **A2**–**A4** were further modified with a chloroacetyl group (ClCH_2_CO‐), a thioacetyl group (HSCH_2_CO‐), and a cysteine [HSCH_2_CH‐(‐NH_2_)‐CO‐], respectively. Moreover, the **B1** backbone, the C‐termini of **B2**–**B7** were further modified with a chloroacetyl derivative [‐NH‐CH(‐CH_2_NHCOCH_2_Cl)‐CO‐; **B2**], a thioacetyl derivative [‐NH‐CH(‐CH_2_NHCOCH_2_SH)‐CO‐; **B3**], a cysteine derivative {‐NH‐CH[‐CH_2_NHCOCH(CH_2_SH)‐NH_2_]‐CO‐; **B4**}, and versions of **B3** with different alkyl chain lengths {‐NH‐CH[−(CH_2_)_n_NHCOCH_2_SH]‐CO‐}; **B5** (*n* = 2), **B6** (*n* = 3), and **B7** (*n* = 4).

The thioacetyl group was employed based on its structural similarity to the chloroacetyl group. In contrast, cysteine was used because it exhibits higher nucleophilicity than the thioacetyl group under neutral pH conditions and is expected to promote disulfide bond formation [35]. In this study, these two types of thiol‐containing components were used to investigate the effects of nucleophilicity and steric structure of the thiol moieties on disulfide bond formation in the present system, and the results are described in Section 3.3. **B5**, **B6**, and **B7** were synthesized to investigate how the distance between the Pyr moiety and the reactive group (thioacetyl group) on the B‐probe side affects excimer formation associated with disulfide bond formation.

The base sequences of the **A** series and **B** series are antiparallel and complementary to the 5′ and 3′ ends of DNA **D1**–**D10** (underlines and dotted underlines in Figure 2B), respectively. **D1**–**D10** are sequences with various numbers of adenines inserted between the sequences complementary to the **A** series and **B** series. Among these, **D3** mimics the RNA sequence of the EGFR mutation exon 19 deletion E746–A750. **D11** mimics the RNA sequence of exon 19. **D12** is a scrambled sequence of **D3**. In **D13**, the 10th A residue in **D3** is replaced with C; thus, **D13** contains a single mismatch at the position closest to the Pyr moiety in the **A** series.

The **A** and **B** series were designed such that, upon hybridization with DNA, their Pyr moieties face each other. Furthermore, the ClCH_2_‐ and HS‐ groups in **A2**–**A4** and **B2**–**B7** are also positioned to face each other, enabling covalent bond formation.

### 3.2 Time‐Dependent Ligation and Excimer Formation of Pyr‐PNA Twin Probe

**FIGURE 3:**
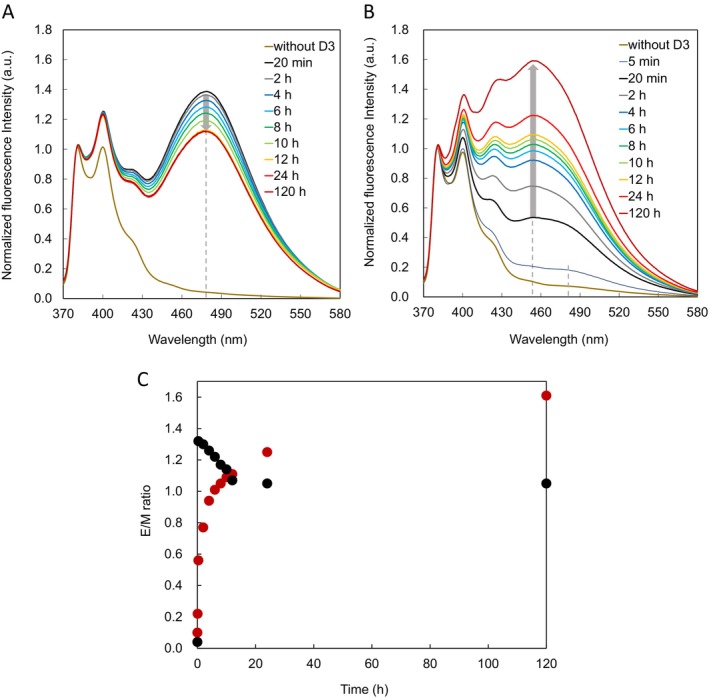
Fluorescence spectra of equimolar mixtures of **A1**/**B1**/**D3** (A) and **A4**/**B3**/**D3** (B) in aqueous buffer at various time points after mixing. (C) Time course of fluorescence intensity of equimolar mixtures of **A1**/**B1**/**D3** (E_480_/M_380_; black circles) and **A4**/**B3**/**D3** (E_455_/M_380_; red circles) based on data shown in (A) and (B).

We first compared fluorescence spectra of PNA twin probe **A1**/**B1** used in our previous study with those of PNA twin probe **A4**/**B3** that is likely to induce ligation (Figures 3 and S5). We measured fluorescence spectra after 20 min of mixing **A1** and **B1** with and without DNA (Figure S5A). In **A1**/**B1** alone, emission from the Pyr monomer was observed at 380 and 400 nm. The same profile was also observed for **A1**/**B1** with non‐complementary **D12**. Meanwhile, in **A1**/**B1** with complementary **D3**, strong emission from the Pyr excimer was observed at 480 nm in addition to the monomer emission. These results indicate that **A1** and **B1** detect a target nucleic acid in a sequence‐specific manner, which is consistent with our previous report on PNA twin probe [27].

Next, we measured fluorescence spectra after 20 min of mixing **A4** and **B3** with and without DNA (Figure S5B). In the fluorescence spectra of **A4**/**B3** with and without **D12**, only monomer‐derived emission was observed. In **A4**/**B3** with **D3**, the emission from the Pyr excimer was observed around 455 nm in addition to the monomer emission, but the excimer emission appeared to be weaker than that observed in **A1**/**B1** with **D3**.

We then measured the fluorescence spectra of the mixtures shown in Figure 3A after 24 h (Figure S5C). All fluorescence spectra showed almost the same profile as those shown in Figure S5A. These are reasonable results, indicating that the fluorescence emission of the mixture remains essentially unchanged over time.

We further measured the fluorescence spectra of the mixtures shown in Figure S5B after 24 h (Figure S5D). The fluorescence spectra of **A4**/**B3** with and without **D12** also showed almost the same profiles as those shown in Figure S5B, which is a reasonable result. In contrast, when **D3** was used, a clear enhancement of excimer fluorescence at 455 nm was observed.

Following these results, we measured the fluorescence spectra of **A1**/**B1** and **A4**/**B3** after incubation with **D3** for various times (Figure 3A,B, respectively). In **A1**/**B1**/**D3**, the fluorescence spectra did not change significantly from 20 min to 120 h, and the excimer fluorescence intensity at 480 nm was observed to be comparable to that of the monomer fluorescence. In contrast, in **A4**/**B3**/**D3**, weak and broad fluorescence signals were observed at 480 and 455 nm after 5 min. An increase in the excimer fluorescence at 455 nm was observed after 20 min, although it remained lower than the monomer emission. The fluorescence intensity further increased over time, and the highest intensity within the observed measurement period was recorded at 120 h. It should be noted that this value does not necessarily represent a plateau or the endpoint of the reaction, as measurements beyond 120 h were not performed.

We plotted the ratio of the fluorescence intensities of the excimer and monomer (E_480_/M_380_, the ratio of 480–380 nm for **A1**/**B1**/**D3**; and E_455_/M_380_, the ratio of 455–380 nm for **A4**/**B3**/**D3**) against time (Figure 3C). Assuming that E_480_/M_380_ (= 0.04) of **A1**/**B1** after 20 min without **D3** is the E_480_/M_380_ of **A1**/**B1**/**D3** after 0 min, E_480_/M_380_ rapidly increased after 20 min, reaching a maximum of 1.32. This indicates that hybridization between Pyr‐PNAs and DNA was completed instantaneously. After that, for unknown reasons, the E_480_/M_380_ gradually decreased, and the E_480_/M_380_ ratio was 1.07 after 12 h, after which it remained almost constant. Although the cause of the gradual decrease in excimer fluorescence is unclear, it cannot be ruled out that aggregation of PNA components or photobleaching of the Pyr moieties may have occurred during the experiment.

In contrast, E_455_/M_380_ of **A4**/**B3**/**D3** gradually increased after mixing, reaching 0.56 after 20 min and 1.11 after 12 h, exceeding that of **A1**/**B1**/**D3**. E_455_/M_380_ continued to increase slowly, reaching 1.25 after 24 h and 1.61 after 120 h. We further measured equimolar mixtures of **A4**/**B3** and **A4**/**B3**/**D3** (final concentration of each was 20 μM) by RP‐HPLC after 24 h (Figure S6). In **A4**/**B3**, the peaks of **A4** and **B3** were observed at 9.50 and 9.25 min, respectively, while in **A4**/**B3**/**D3**, a new broad peak at 10.98 min was confirmed in addition to these peaks. Fractions of the new peak were collected, and MALDI‐TOF mass spectrometry was performed (Figure S7), indicating that **A4** and **B3** were linked via a disulfide bond (observed: 7750.19 Da, calculated: 7752.33 Da).

In Figure 3, the excimer fluorescence of **A4**/**B3**/**D3** (455 nm) is blue‐shifted by 25 nm compared with that of **A1**/**B1**/**D3** (480 nm). We initially considered that this shift might reflect a transition from a dynamic to a static excimer, as reported for pyrene systems [36, 37, 38, 39, 40]. However, analysis of the excitation and UV–vis spectra (Figure S8) indicates that both emissions originate from dynamic excimers. Therefore, the observed blue shift is more likely attributable to differences in the relative orientation and distance between the two Pyr moieties. In particular, covalent linkage via disulfide bond formation is expected to restrict conformational flexibility and impose a more defined geometry on the Pyr pair. Such structural constraints can alter the excimer emission energy, resulting in a blue shift.

These results indicate that the PNA twin probe **A4**/**B3** can detect target DNA by fluorescence from stable excimers formed over time. This fluorescence occurs through ligation caused by reactive groups contained in the two Pyr‐PNAs after hybridization to DNA. Furthermore, the fluorescence intensity was found to be higher than that of the unlinked PNA twin probe reported previously.

### 3.3 Effect of Terminal Reactive Groups on Ligation and Excimer Formation

**FIGURE 4:**
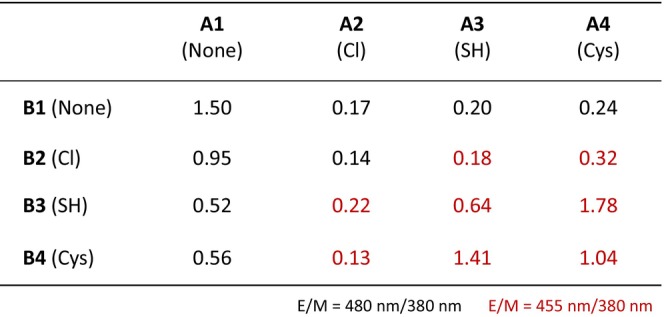
E/M values obtained from the fluorescence spectra shown in Figure S9. Black and red numbers indicate E_480_/M_380_ and E_455_/M_380_, respectively.

Based on the concept shown in Figure 1, if one of the two Pyr‐PNAs does not have a reactive group, the ligation will not proceed. Meanwhile, the ligation may also proceed with other pairs of reactive groups, and excimer fluorescence may be obtained via a process similar to that of **A4**/**B3**. Therefore, we measured the fluorescence spectra of the mixtures containing **D3** with combinations of **A1**–**A4** and **B1**–**B4** after 120 h (Figure S9). The E_480_/M_380_ and E_455_/M_380_ values obtained from these fluorescence spectra are summarized in Figure 4.

Again, **A1**/**B1** (none/none) showed an E_480_/M_380_ value of 1.50 (Figure S9A). **A1**/**B2** (none/chloroacetyl), **A1**/**B3** (none/thioacetyl), and **A1**/**B4** (none/Cys) exhibited lower E_480_/M_380_ values (0.95, 0.52, and 0.56, respectively), indicating that excimer formation via ligation does not occur in the absence of a reactive group and that such modifications may sterically interfere with excimer formation between the two Pyr‐PNAs on the DNA template.

Similar trends were observed for **A2**/**B1** (chloroacetyl/none), **A3**/**B1** (thioacetyl/none), and **A4**/**B1** (Cys/none), as shown in Figure S9B, with E_480_/M_380_ values of 0.17, 0.20, and 0.24, respectively. The stronger decrease observed in the **B**‐series modifications compared with the **A**‐series remains unclear, but in any case, the reactive‐group modifications that do not support ligation negatively affect excimer formation.

**A2**/**B2** (chloroacetyl/chloroacetyl) is also not expected to support ligation, and as expected, showed a smaller E_480_/M_380_ value (0.14), as shown in Figure S9C. Meanwhile, in the case of **A2**/**B3** (chloroacetyl/thioacetyl), **A2**/**B4** (chloroacetyl/Cys), **A3**/**B2** (thioacetyl/chloroacetyl), and **A4**/**B2** (Cys/chloroacetyl), we expected that, when two Pyr‐PNAs formed a hybrid on **D3**, the chloroacetyl group would react with the thiol group to form a thioether bond. However, unfortunately, E_455_/M_380_ showed small values of 0.22, 0.13, 0.18, and 0.32, respectively. These results suggest that the reaction of the thiol group with the chloroacetyl group hardly proceeds under these experimental conditions. These findings are also consistent with the results in Figure S9A,B that the reactive groups sterically inhibit excimer formation. However, we note that slight excimer fluorescence was observed at 455 nm for **A4**/**B2**. We speculate that this emission represents excimer fluorescence via the formation of a thioether bond.

Next, disulfide bond formation was expected in **A3**/**B3**, **A3**/**B4**, **A4**/**B3**, and **A4**/**B4**, which are modified with thiol groups (Figure S9D). Among these combinations, **A4**/**B3** (Cys/thioacetyl) showed the highest E_455_/M_380_ value (1.78), followed by **A3**/**B4** (thioacetyl/Cys, 1.41). In addition, **A3**/**B3** (thioacetyl/thioacetyl) and **A4**/**B4** (Cys/Cys) showed lower values of 0.64 and 1.04, respectively. Although Cys was expected to promote disulfide bond formation and excimer emission more efficiently than the thioacetyl group due to its higher nucleophilicity, the observed trends suggest that both the combination of reactive groups and their steric and structural factors collectively influence disulfide bond formation and excimer emission [35].

### 3.4 Effect of Reactive Group Distance on Ligation and Excimer Formation

**FIGURE 5:**
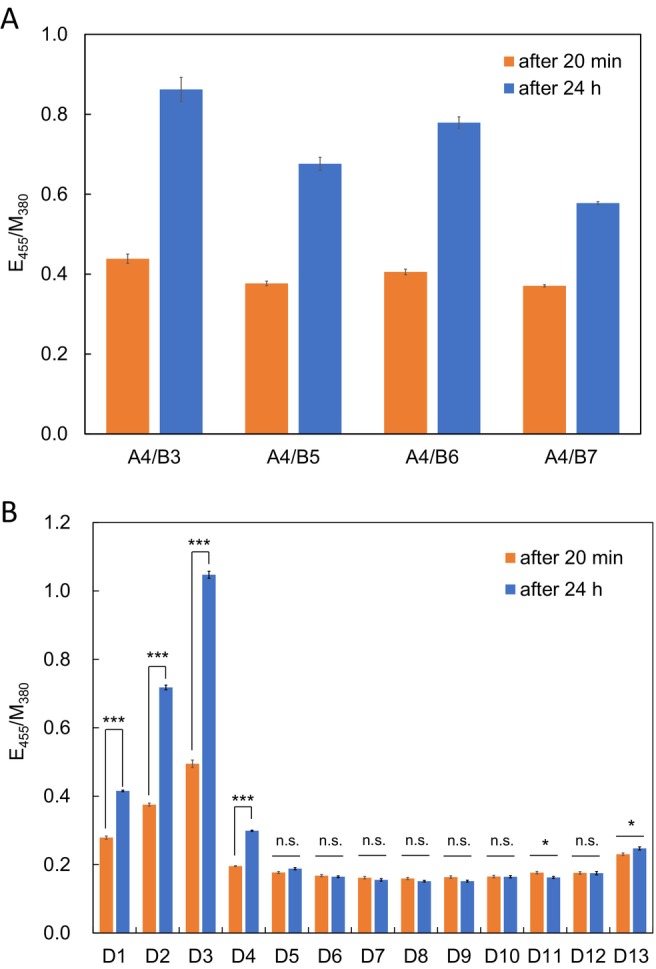
E_455_/M_380_ obtained from fluorescence spectra after 20 min and 24 h for equimolar mixtures of (A) **A4** and **B3** or **B5**–**B7** with **D3** and (B) **A4** and **B3** with **D1**–**D13**. Fluorescence intensity of complex‐derived signals was quantified from fluorescence spectra (mean ± SD, *n* = 4, *p < 0.05, ***p < 0.001). Statistical significance was evaluated using one‐way ANOVA followed by Tukey's multiple comparisons test.

Next, to assess whether the distance of the reactive groups in the Pyr‐PNAs on the DNA template affects the formation of excimer through ligation, we measured the fluorescence spectra of equimolar mixtures of **A4**/**B3** with different linker lengths between the PNA and the reactive group in aqueous solutions containing **D3** after 20 min and 24 h, and compared their E_455_/M_380_ (Figures 5A and S10).

All **A4**/**B3** and **A4**/**B5**–**B7** showed higher E_455_/M_380_ after 24 h than after 20 min, indicating that ligation proceeds and excimer fluorescence increases. Meanwhile, it was also revealed that the difference in linker length affects E_455_/M_380_ after 24 h. E_455_/M_380_ reached a maximum of 0.86 for **A4**/**B3** bearing Dap(‐COCH_2_SH) and a minimum of 0.58 for **A4**/**B7** with Lys(‐COCH_2_SH). That is, within this measurement range, longer alkyl linkers tended to show smaller E_455_/M_380_. This result indicates that the difference in alkyl linker chain length affects the promotion of the reaction between reactive groups, which is reflected in the excimer fluorescence through ligation. In other words, this result indicates that there is an appropriate distance between reactive groups for ligation to occur.

Next, we assessed E_455_/M_380_ after 20 min and 24 h for **A4**/**B3**, containing DNA **D1**–**D11** with different numbers of inserted adenine residues (Figures 5B and S11). For **D1**, **D2**, and **D3**, E_455_/M_380_ increased with time, and the values increased as the number of inserted adenine residues increased (0.42, 0.72, and 1.05 after 24 h, respectively). However, E_455_/M_380_ for **D4** decreased significantly (0.30 after 24 h), and E_455_/M_380_ for **D5** and later remained decreased and showed almost no change at 20 min (0.16–0.18) and 24 h (0.15–0.19). In other words, E_455_/M_380_ decreased as the number of inserted adenine residues increased. This result was not limited to the inserted base being adenine, but was the same for **D11** (0.18 after 20 min, 0.16 after 24 h). These results clearly indicate that the linkage of the reactive groups between the Pyr‐PNAs on DNA is affected by these distances, and furthermore, that there is an optimal distance. Among the DNAs used in this study, **D3** provides the optimal distance for the linkage. We speculate that, in **D2** and **D1**, the Pyr‐PNAs are too close to each other and, as a result, the reactive groups are separated from each other. We also speculate that when Pyr‐PNAs are separated by four or more bases along the DNA, the reactive groups cannot interact, and linkage does not occur.

Again, **A4**/**B3** with the scrambled sequence **D12** showed no excimer fluorescence response regardless of time (E_455_/M_380_ was 0.18 after 20 min and 24 h). Under these conditions, **A4** and **B3** are not expected to form a hybrid with **D12** in the first place, indicating that the binding of **A4** and **B3** is essential for their assembly on the DNA template.

**D13** corresponds to the **D3** sequence containing one mismatch at the position closest to the Pyr of **A4**. Both **A4** and **B3** are expected to form hybrids with **D13**, but E_455_/M_380_ was almost unchanged after 20 min (0.23) and 24 h (0.25), implying that a fluctuation of reactive groups caused by the mismatch at the terminus of Pyr‐PNA significantly inhibits ligation.

These results indicate that, in PNA twin probes, adjusting the distance between the reactive groups of Pyr‐PNAs on DNA is important for the reaction between the reactive groups and subsequent stable excimer formation and fluorescence emission via ligation.

### 3.5 Intracellular Fluorescence Detection of Target RNA Using Reactive Pyr‐PNA Twin Probe

**FIGURE 6:**
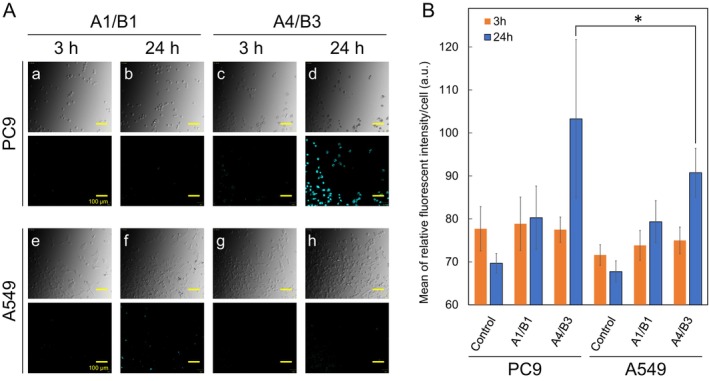
(A) Fluorescence images from live‐cell imaging of PC9 or A549 cells incubated with the PNA twin probe. The upper panels show bright‐field images and the lower panels show fluorescence images. The PNA twin probe was used at an incubation concentration of 10 μM, and images were acquired at 3 and 24 h after probe addition. The scale bar represents 100 μm. (B) Quantification of complex‐derived fluorescence intensity obtained from the fluorescence images (mean ± sd, *n* = 4, *p < 0.05). Fluorescence intensity was quantified at the single‐cell level, and the mean value within each well was calculated and treated as one independent sample (*n* = 1). Four independent wells were analyzed per condition. Statistical significance was determined using one‐way ANOVA followed by Tukey's multiple comparisons test. In the figure, “Control” indicates cells without the PNA twin probe (negative control).

Finally, to examine whether **A4**/**B3** detects target RNA in living cells, lung adenocarcinoma cell lines PC9 and A549 were incubated with the probe and observed by fluorescence microscopy (Figure 6). **A4** and **B3** contain a sequence complementary to the sequence caused by a deletion mutation in exon 19 of EGFR (E19D) expressed in PC9 cells (5′‐GUCGCUAUCAAAACAUCUCCGA‐3′; the solid and dashed underlines are the sequences complementary to **A4** and **B3**, respectively; the RNA sequence corresponds to **D3**). Meanwhile, **A4**/**B3** also contains a sequence complementary to the sequence of exon 19 of normal EGFR (E19) expressed in A549 cells (5′‐GUCGCUAUCAAGGAAUUAAGAGAAGCAACAUCUCCGA‐3′; the RNA sequence corresponds to **D11**). However, as shown by in vitro experiments, excimer fluorescence is expected to occur at E19D but not at E19 due to differences in base insertion between the hybrids formed with RNA by **A4** and **B3**.

Figures 6A and S12 show bright‐field images (top panels) and fluorescence images (bottom panels) of PC9 and A549 cells treated for 3 or 24 h with either the non‐reactive **A1**/**B1** or the reactive **A4**/**B3** at the concentration of 1 and 10 μM. In PC9 cells treated with **A1**/**B1** and 1 μM of **A4**/**B3**, no intracellular emission indicative of pyrene excimer fluorescence was observed under either condition after 3 h (panel a) or 24 h (panel b). In contrast, in PC9 cells treated with 10 μM of **A4**/**B3**, excimer fluorescence was not detected after 3 h (panel c), whereas clear intracellular excimer fluorescence was observed after 24 h (panel d). Similarly, in A549 cells, no excimer emission was observed under either condition with **A1**/**B1** or **A4**/**B3** (panels e–h). To validate that the observed fluorescent signals were emitted by the excimer formed probes, PC9 cells were treated with either the **A4** alone or the **B3** alone under the same experimental conditions used for the **A4/B3** combination. In these control experiments, strong fluorescence signals were not observed (Figure S13). These results strongly indicate that the observed fluorescence signals were derived from pyrene excimer emission rather than from monomer emission or nonspecific probe effects. Figure 6B shows the intracellular fluorescence intensity calculated from these fluorescence images. For **A1**/**B1**, fluorescence was barely detectable in either PC9 or A549 cells. In contrast, **A4**/**B3** exhibited significantly higher excimer fluorescence in PC9 cells after 24 h compared with A549 cells. The high background fluorescence may be attributed to nonspecific cooperative effects, such as local probe accumulation and homodimerization of the PNA probes.

Finally, cytotoxic effects of the **A4/B3** were validated using a FITC–Annexin V assay system after treatment at 10 μM for 24 h. As shown in Figure S14, Annexin V–positive staining was observed, particularly in PC9 cells, whereas A549 cells exhibited comparatively weaker cytotoxic responses. These results suggest that the observed cytotoxicity is cell line–dependent rather than nonspecific. The PC9 cells are known to be strongly dependent on EGFR signaling for survival compared to A549 cells. Thus, it is plausible that the **A4/B3** exerts cytotoxic effects in PC9 cells, at least in part, through partial interference with EGFR signaling; however, the precise underlying mechanism remains to be elucidated in future.

These results suggest that, under the conditions of this study, in PC9 cells containing the target RNA sequence, excimer fluorescence expected from **A1**/**B1** is not sufficiently observed, whereas **A4**/**B3**, consistent with in vitro results, gradually produces excimer fluorescence exceeding that of **A1**/**B1**, enabling the fluorescent detection of the target RNA within the cells.

## 4 Conclusions

In this study, we successfully detected target nucleic acids via excimer fluorescence using a pair of thiol‐containing Pyr‐PNAs. The observed excimer fluorescence arises from the proximity of thiol groups induced by hybridization of the Pyr‐PNAs on a DNA template, followed by ligation via disulfide bond formation. In the unreacted state, excimer formation is sterically hindered by the presence of free thiol moieties. However, as ligation proceeds and thiols are converted into disulfide bonds, this steric restriction is released, and the relative orientation of the Pyr moieties becomes more fixed, thereby promoting stable excimer formation and enhanced fluorescence emission.

Compared with the conventional PNA twin probe (**A1**/**B1**), the reactive PNA twin probe (**A4**/**B3**) exhibited time‐dependent and ligation‐dependent excimer formation, resulting in significantly enhanced fluorescence intensity. These results indicate that covalent linkage plays a crucial role in stabilizing the excimer state and improving signal output. Furthermore, ligation efficiency, as well as the resulting excimer formation and fluorescence emission, was found to depend on the inter‐site distance of the PNA twin probe on the DNA template, suggesting that precise spatial arrangement is critical for optimal performance. Notably, the present PNA twin probe also enabled fluorescence‐based detection of target RNA in living cells, demonstrating its potential applicability in biological environments.

To place these findings in context, conventional PNA fluorescent probes typically employ a single PNA strand and detect nucleic acids mainly through fluorescence on/off switching. Similarly, molecular beacons utilize a single PNA strand, where target recognition is based on either a fluorescence turn‐on response or acceptor emission resulting from FRET disruption. In contrast, the PNA twin probe developed in this study employs two PNA strands, thereby increasing the number of recognition bases and enabling higher sequence specificity. In addition, although optimization of fluorophore distance and orientation is required, this system enables excimer formation and FRET‐related processes, allowing ratiometric detection based on longer‐wavelength fluorescence signals. A further distinctive feature of the reactive PNA probe is that the two PNA strands are covalently linked after hybridization. This structural fixation is expected to further stabilize the emissive state and enhance fluorescence output.

Finally, we consider that further investigation is required regarding the emission behavior of the PNA twin probe after dissociation from the RNA/PNA hybrid. If the disulfide‐linked PNA twin probe retains its emissive state after dissociation, it may enable sequential generation of fluorescent probes from a single RNA target, potentially leading to signal amplification. Moreover, although not fully explored in this study, the development of reactive PNA twin probes based on thioether bond formation remains an important future direction. Given the reducing intracellular environment, thioether bonds are expected to exhibit higher stability than disulfide bonds, which may further enhance signal stability and amplification after reaction.

## Author Contributions

**Yutaka Ouchi:** data curation, investigation, visualization. **Koki Ishii:** methodology, investigation, visualization. **Yumiko Sato:** data curation, visualization. **Yoshitane Imai:** funding acquisition. **Hideo Matsui:** software, writing – review and editing. **Takashi Ohtsuki:** funding acquisition. **Yoshiyuki Hakata:** investigation, resources. **Hajime Shigeto:** data curation, formal analysis, investigation, validation, writing – review and editing. **Shohei Yamamura:** funding acquisition, resources. **Mizuki Kitamatsu:** conceptualization, funding acquisition, methodology, project administration, supervision, visualization, writing – original draft.

## Funding

This work was supported by Kindai University (IP009).

## Conflicts of Interest

The authors declare no conflicts of interest.

## Supporting information


**Figure S1:** MALDI‐TOF mass spectra of A1–A4. α‐CHCA was used as a matrix. Calcd. [M + H]^+^: A1 = 3773.70, A2 = 3849.67, A3 = 3847.68, A4 = 3875.81; Obsd. [M + H]^+^: A1 = 3773.06, A2 = 3849.96, A3 = 3847.32, A4 = 3876.03.
**Figure S2:** MALDI‐TOF mass spectra of B1–B7. α‐CHCA was used as a matrix. Calcd. [M + H]^+^: B1 = 3677.60, B2 = 3881.63, B3 = 3879.64, B4 = 3908.66, B5 = 3893.65, B6 = 3907.67, and B7 = 3921.68; Obsd. [M + H]^+^: B1 = 3678.44, B2 = 3882.10, B3 = 3881.02, B4 = 3908.16, B5 = 3894.71, B6 = 3906.90, and B7 = 3922.65.
**Figure S3:** RP‐HPLC chromatograms of A1–A4. Eluent A, 0.1% TFA in water; Eluent B, acetonitrile. Monitoring at 340 nm with a gradient of 0%–80% eluent B over 20 min. Purities: A1 (82.4%), A2 (100%), A3 (100%), and A4 (100%).
**Figure S4:** RP‐HPLC chromatograms of B1–B7. Eluent A, 0.1% TFA in water; Eluent B, acetonitrile and monitoring at 340 nm with a gradient of 0%–80% eluent B over 20 min. Purities: B1 (100%), B2 (100%), B3 (91.2%), B4 (100%), B5 (94.0%), B6 (94.8%), and B7 (100%).
**Figure S5:** Fluorescence spectra of equimolar mixtures of A1/B1 (black solid line), A1/B1/D3 (red solid line), and A1/B1/D12 (black dotted line) after 20 min (A) and 24 h (C) in aqueous buffer solution. Fluorescence spectra of equimolar mixtures of A4/B3 (black solid line), A4/B3/D3 (red solid line), and A4/B3/D12 (black dotted lines) after 20 min (B) and 24 h (D) in aqueous buffer solution.
**Figure S6:** RP‐HPLC chromatograms of equimolar mixtures of A4/B3 and A4/B3/D3 after 12 h. Eluent A, 0.1% TFA in water; Eluent B, acetonitrile; monitoring at 340 nm with a gradient of 10%–80% eluent B over 20 min.
**Figure S7:** MALDI‐TOF mass spectrum of a fractionated sample of the peak at 10.98 min shown by HPLC of an equimolar mixture of A4/B3/D3 after 24 h (Figure S5). α‐CHCA was used as a matrix. The peak at 3877.57 corresponds to a doubly charged peak at 7752.16.
**Figure S8:** (A) UV–vis spectra of the equimolar mixture of A1/B1/D3 (black line) and the equimolar mixture of A4/B3/D3 (red line) after 120 h. These measurements were conducted with a JASCO V‐560 UV–vis spectrometer and a 1 cm quartz cell at wavelengths from 200 to 800 nm. Each of the final concentrations was 10 μM. The peaks derived from Pyr at 335 and 350 nm are confirmed. (B) Excitation spectra of A1/B1/D3 and (C) A4/B3/D3. Emission wavelengths were 380 nm (dotted lines) and 480 nm (black solid line) or 455 nm (red solid line). These excitation spectra were measured at excitation wavelengths from 280 to 460 nm. The final concentration of each sample was 10 μM. All of these measurements were carried out in aqueous solution (10 mM PBS, pH 7.0) at 25°C.
**Figure S9:** (A–D) Fluorescence spectra of equimolar mixtures of A1–A4, B1–B4, and D3 in aqueous buffer solution (10 mM PBS, pH 7.0). Fluorescence spectra were measured 120 h after mixing at 25°C. The final concentration of each of the A series, B series, and D3 was 1.0 μM. Excitation wavelength was 350 nm.
**Figure S10:** Fluorescence spectra of equimolar mixtures of (A) A4/B3/D3, (B) A4/B5/D3, (C) A4/B6/D3, and (D) A4/B7/D3 after various times. (E) Time course of E_455_/M_380_ ratio of equimolar mixtures of A4/B3/D3, A4/B5/D3, A4/B6/D3, and A4/B7/D3 based on (A–D).
**Figure S11:** Fluorescence spectra of equimolar mixtures of A4/B3/D1–D13 after 20 min (black lines) and 24 h (red lines).
**Figure S12:** Fluorescence images of live PC9 or A549 cells incubated with the 10 μM concentration of only A4 or only B3 probes. The images were acquired at 3 and 24 h after probe addition. The scale bar represents 100 μm.
**Figure S13:** Fluorescence images of live PC9 or A549 cells incubated with the 1 μM concentration of PNA twin probe. The images were acquired at 3 and 24 h after probe addition. The scale bar represents 100 μm.
**Figure S14:** Fluorescence images of apoptosis assay using FITC‐Annexin V 24 h after treatment of 10 μM concentration of A4/B3 twin probe. The scale bar represents 100 μm.

## Data Availability

The data supporting the findings of this study are available from the corresponding author upon reasonable request.
