# Supplementary material for: Detection of a Target Nucleic Acid by Ligation‐Assisted Fluorescence Enhancement of a Peptide Nucleic Acid (PNA) Twin Probe via Disulfide Binding
Source: Biopolymers. 2026 May 15;117:e70105. doi: 10.1002/bip.70105 (PMC13178207; doi:10.1002/bip.70105)
Supplement: Supplementary file 1 — Figure S1: MALDI‐TOF mass spectra of A1–A4. α‐CHCA was used as a matrix. Calcd. [M + H]+: A1 = 3773.70, A2 = 3849.67, A3 = 3847.68, A4 = 3875.81; Obsd. [M + H]+: A1 = 3773.06, A2 = 3849.96, A3 = 3847.32, A4 = 3876.03. Figure S2: MALDI‐TOF mass spectra of B1–B7. α‐CHCA was used as a matrix. Calcd. [M + H]+: B1 = 3677.60, B2 = 3881.63, B3 = 3879.64, B4 = 3908.66, B5 = 3893.65, B6 = 3907.67, and B7 = 3921.68; Obsd. [M + H]+: B1 = 3678.44, B2 = 3882.10, B3 = 3881.02, B4 = 3908.16, B5 = 3894.71, B6 = 3906.90, and B7 = 3922.65. Figure S3: RP‐HPLC chromatograms of A1–A4. Eluent A, 0.1% TFA in water; Eluent B, acetonitrile. Monitoring at 340 nm with a gradient of 0%–80% eluent B over 20 min. Purities: A1 (82.4%), A2 (100%), A3 (100%), and A4 (100%). Figure S4: RP‐HPLC chromatograms of B1–B7. Eluent A, 0.1% TFA in water; Eluent B, acetonitrile and monitoring at 340 nm with a gradient of 0%–80% eluent B over 20 min. Purities: B1 (100%), B2 (100%), B3 (91.2%), B4 (100%), B5 (94.0%), B6 (94.8%), and B7 (100%). Figure S5: Fluorescence spectra of equimolar mixtures of A1/B1 (black solid line), A1/B1/D3 (red solid line), and A1/B1/D12 (black dotted line) after 20 min (A) and 24 h (C) in aqueous buffer solution. Fluorescence spectra of equimolar mixtures of A4/B3 (black solid line), A4/B3/D3 (red solid line), and A4/B3/D12 (black dotted lines) after 20 min (B) and 24 h (D) in aqueous buffer solution. Figure S6: RP‐HPLC chromatograms of equimolar mixtures of A4/B3 and A4/B3/D3 after 12 h. Eluent A, 0.1% TFA in water; Eluent B, acetonitrile; monitoring at 340 nm with a gradient of 10%–80% eluent B over 20 min. Figure S7: MALDI‐TOF mass spectrum of a fractionated sample of the peak at 10.98 min shown by HPLC of an equimolar mixture of A4/B3/D3 after 24 h (Figure S5). α‐CHCA was used as a matrix. The peak at 3877.57 corresponds to a doubly charged peak at 7752.16. Figure S8: (A) UV–vis spectra of the equimolar mixture of A1/B1/D3 (black line) and the equimolar mixture of A4/B3/D3 (red [file BIP-117-e70105-s001.docx]

Supporting Information

Detection of a target nucleic acid by ligation-assisted fluorescence enhancement of a peptide nucleic acid (PNA) twin probe via disulfide binding

Yutaka Ouchi, Koki Ishii, Yumiko Sato, Yoshitane Imai, Hideo Matsui, Takashi Ohtsuki, Yoshiyuki Hakata, Hajime Shigeto, Shohei Yamamura, and Mizuki Kitamatsu

Table of contents

Fig. S1. MALDI-TOF mass spectra of **A1**–**A4**.

Fig. S2. MALDI-TOF mass spectra of **B1**–**B7**.

Fig. S3. HPLC chromatograms of **A1**–**A4**.

Fig. S4. HPLC chromatograms of **B1**–**B7**.

Fig. S5. Fluorescence spectra of **An**/**Bn**, **An**/**Bn**/**D3**, and **An**/**Bn**/**D12** (**n** = 1–4).

Fig. S6. HPLC chromatograms of **A4**/**B3** and **A4**/**B3**/**D3**.

Fig. S7. MALDI-TOF mass spectrum of **A4**/**B3**/**D3**.

Fig. S8. UV and Excitation spectra of **A1**/**B1**/**D3** and **A4**/**B3**/**D3**.

Fig. S9. Fluorescence spectra of **A1**–**A4**, **B1**–**B4**, and **D3**.

Fig. S10. Fluorescence spectra of **A4**/**B3**/**D3**, **A4**/**B5**/**D3**, **A4**/**B6**/**D3**, and **A4**/**B7**/**D3**.

Fig. S11. Fluorescence spectra of **A4**/**B3**/**D1–D13**.

Fig. S12. Fluorescence images from cells incubated with **A4** or **B3**.

Fig. S13. Fluorescence images from cells incubated with 1 μM **A1**/**B1** or **A4**/**B3**.

Fig. S14. FITC-Annexin V apoptosis assay images after **A4**/**B3** treatment.

**
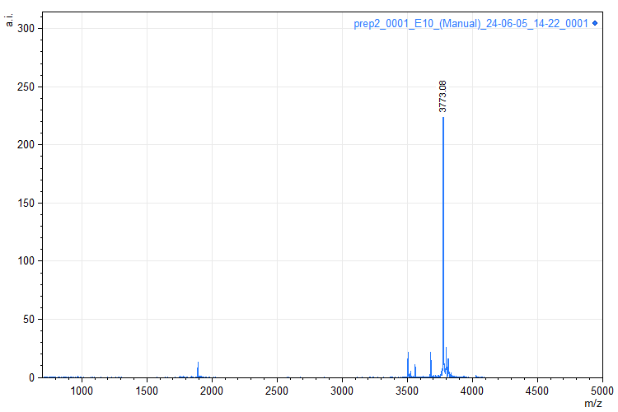
**

**A1**

**
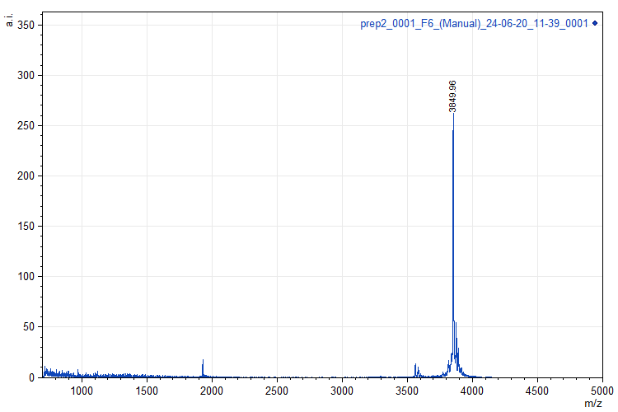
**

**A2**


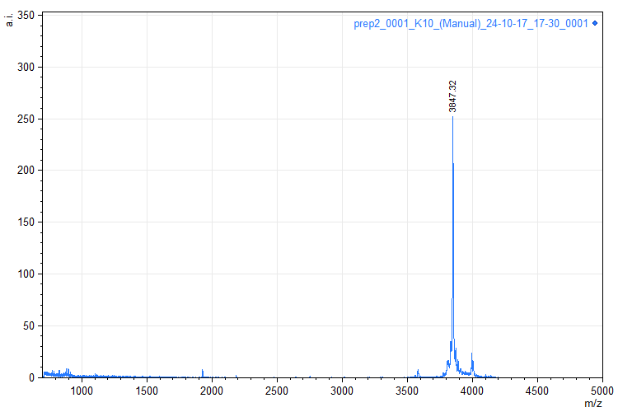


**A3**

**
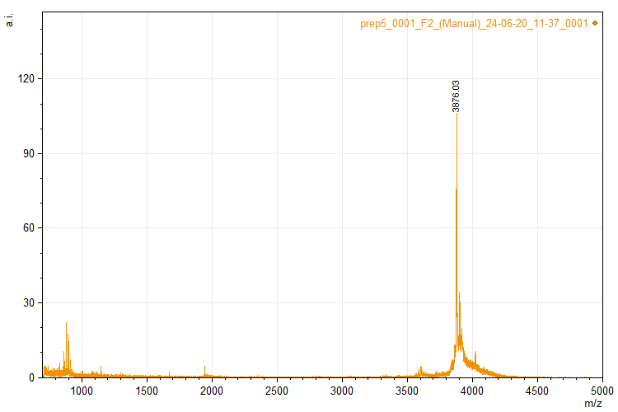
**

**A4**

**Fig. S1.** MALDI-TOF mass spectra of **A1**–**A4**. α-CHCA was used as a matrix. Calcd. [M+H]^+^: **A1** = 3773.70, **A2** = 3849.67, **A3** = 3847.68, **A4** = 3875.81; Obsd. [M+H]^+^: **A1** = 3773.06, **A2** = 3849.96, **A3** = 3847.32, **A4** = 3876.03.

**
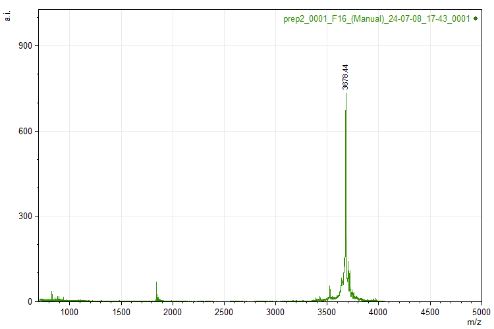
**

**B1**

**
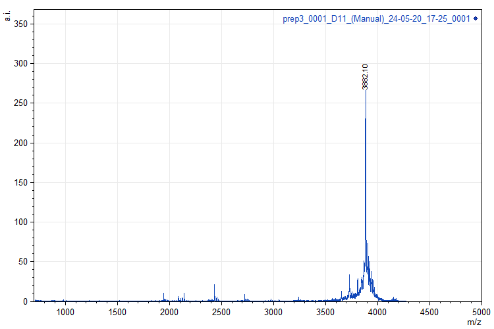
**

**B2**

**
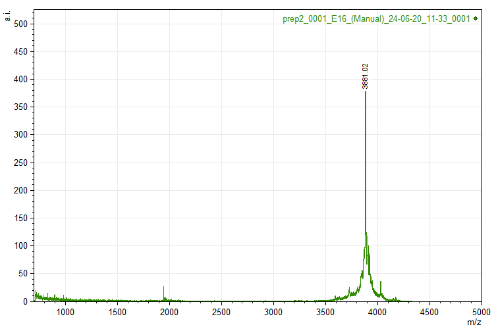
**

**B3**

**
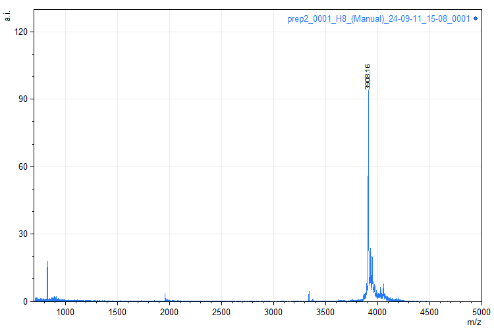
**

**B4**

**
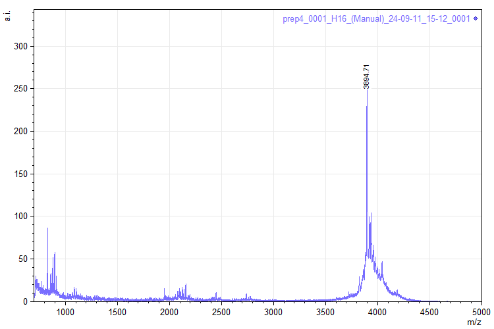
**

**B5**

**
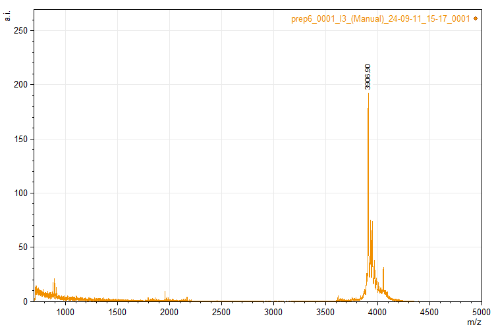
**

**B6**

**
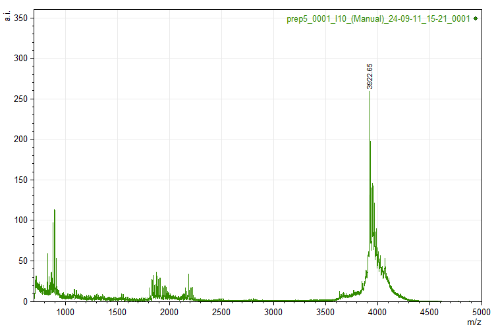
**

**B7**

**Fig. S2.** MALDI-TOF mass spectra of **B1**–**B7**. α-CHCA was used as a matrix. Calcd. [M+H]^+^: **B1** = 3677.60, **B2** = 3881.63, **B3** = 3879.64, **B4** = 3908.66, **B5** = 3893.65, **B6** = 3907.67, and **B7** = 3921.68; Obsd. [M+H]^+^: **B1** = 3678.44, **B2** = 3882.10, **B3** = 3881.02, **B4** = 3908.16, **B5** = 3894.71, **B6** = 3906.90, and **B7** = 3922.65.

**
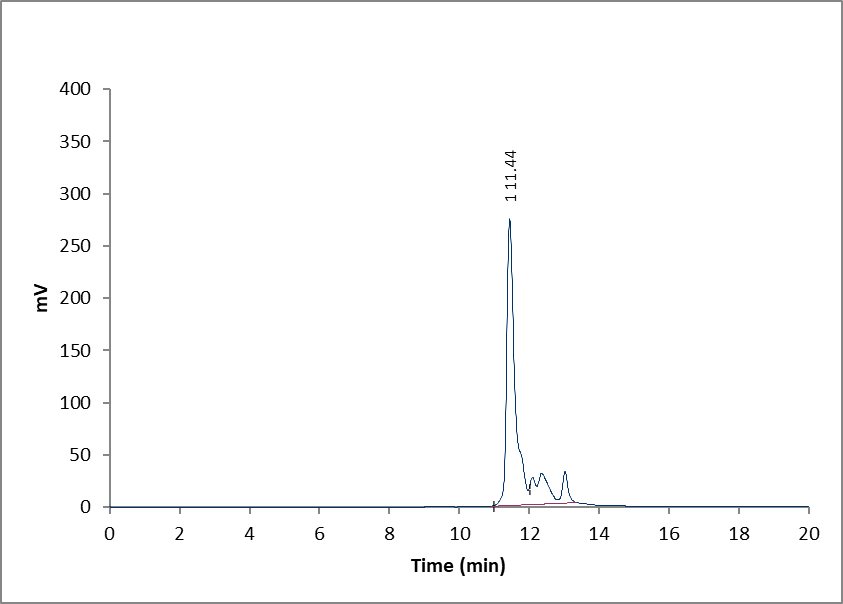
**

**A1**

**
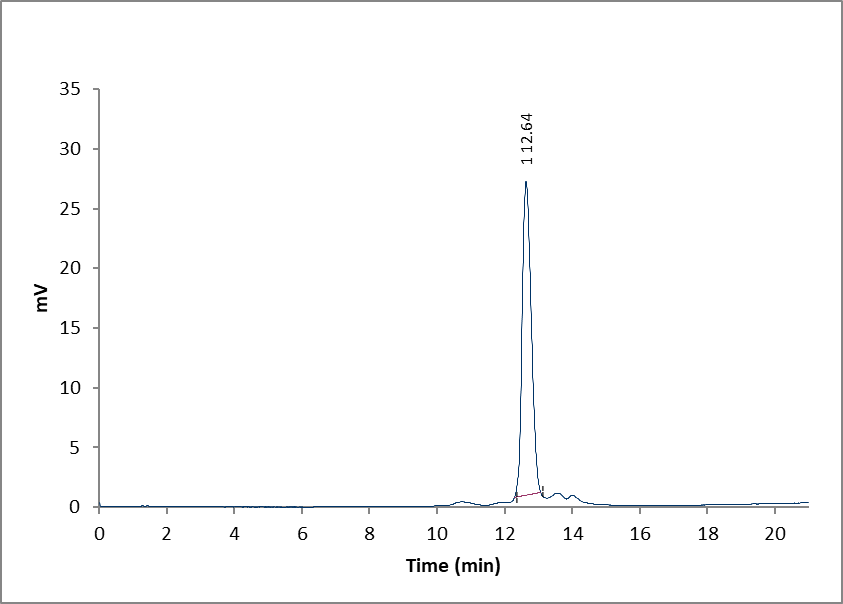
**

**A2**

**
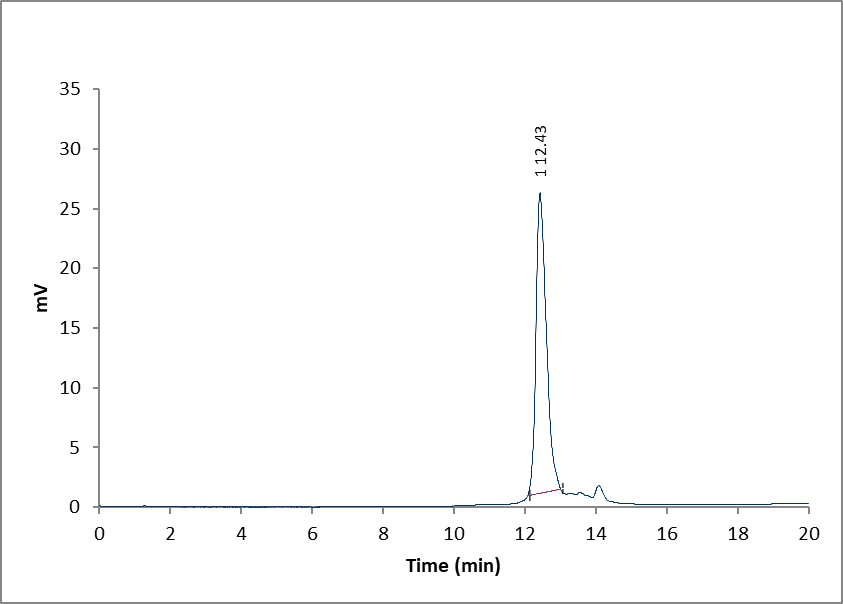
**

**A3**

**
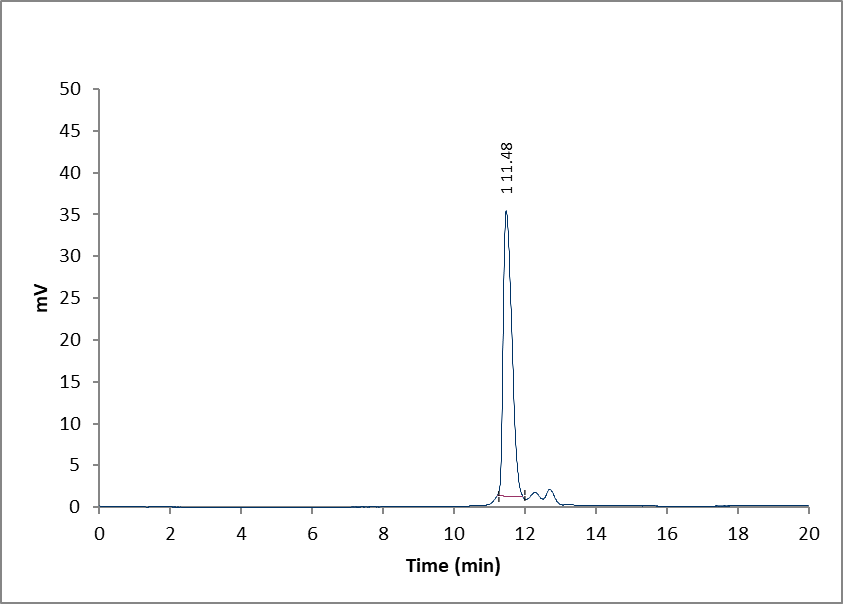
**

**A4**

**Fig. S3.** RP-HPLC chromatograms of **A1**–**A4**. Eluent A, 0.1% TFA in water; Eluent B, acetonitrile. Monitoring at 340 nm with a gradient of 0%–80% eluent B over 20 min. Purities: **A1** (82.4%), **A2** (100%), **A3** (100%), and **A4** (100%).

**
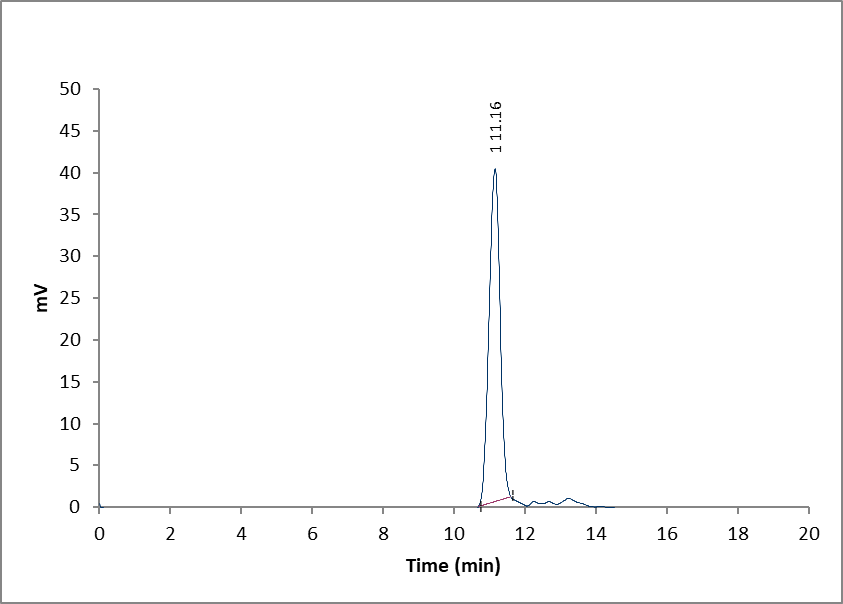
**

**B1**

**
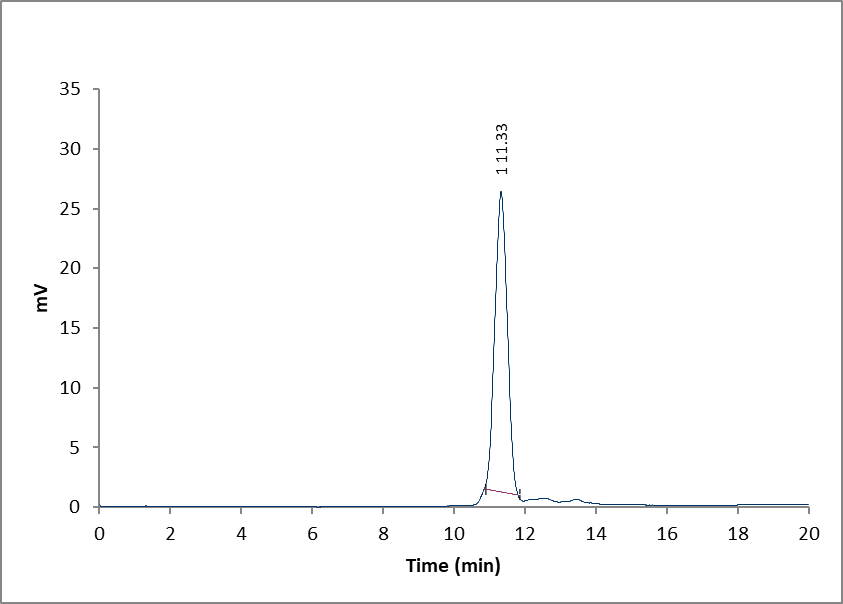
**

**B2**

**
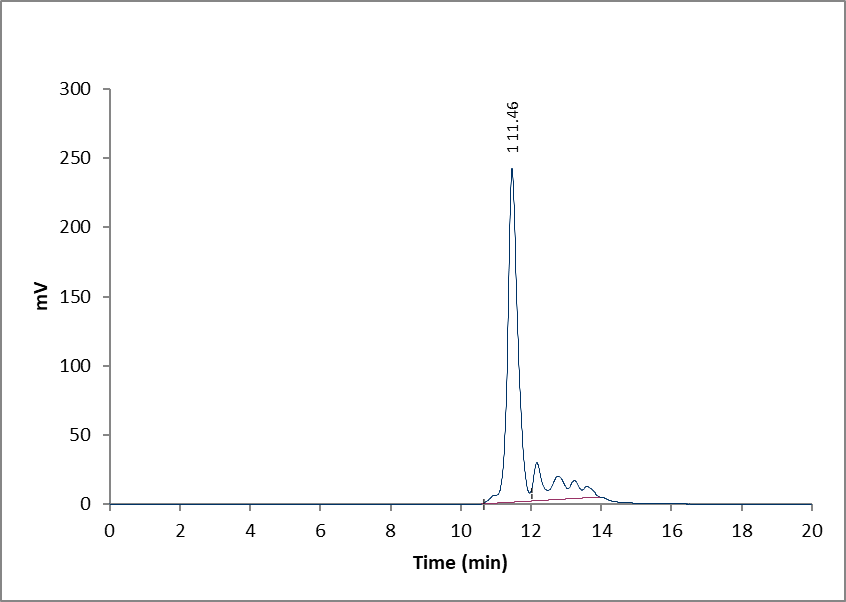
**

**B3**

**
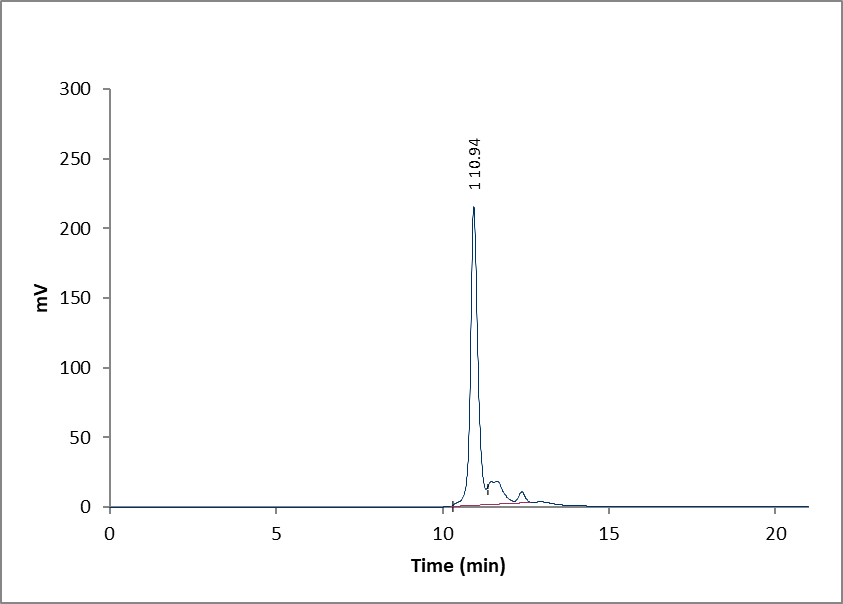
**

**B4**

**
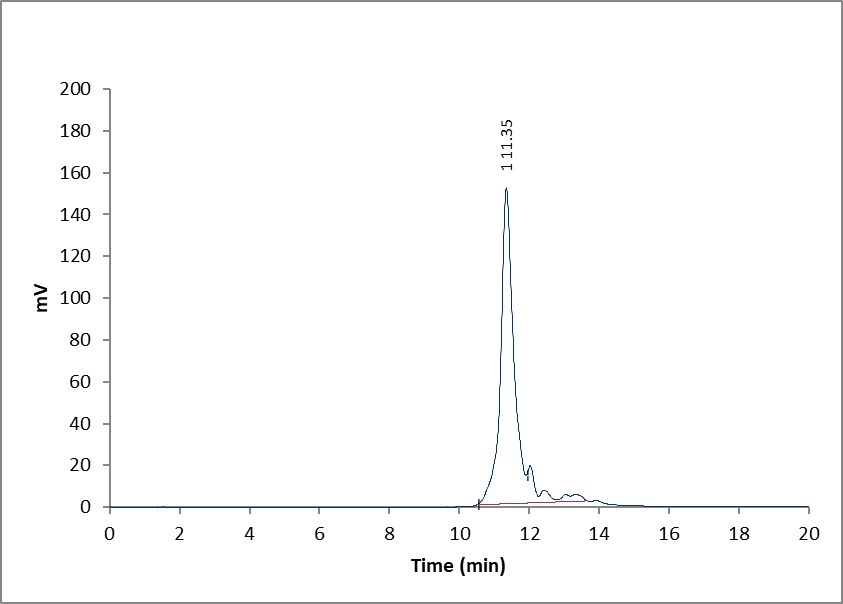
**

**B5**

**
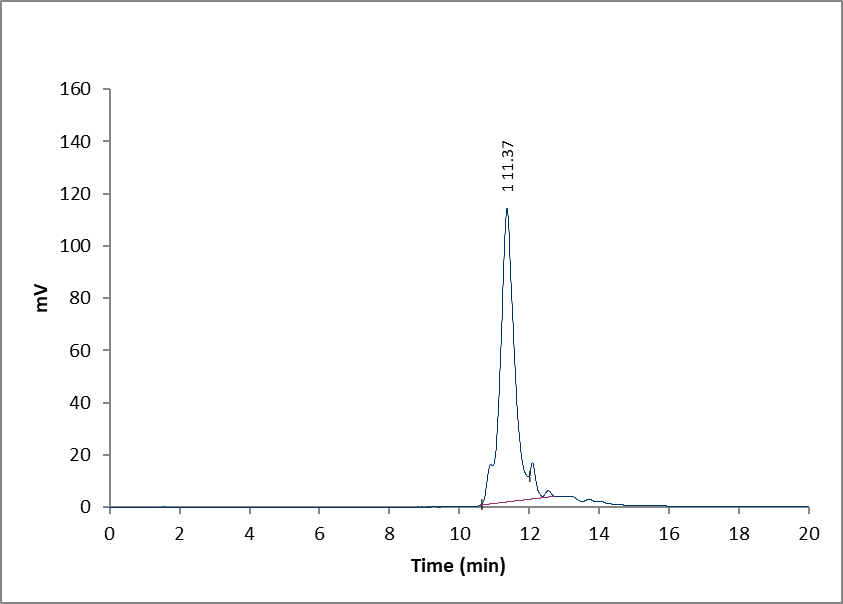
**

**B6**

**
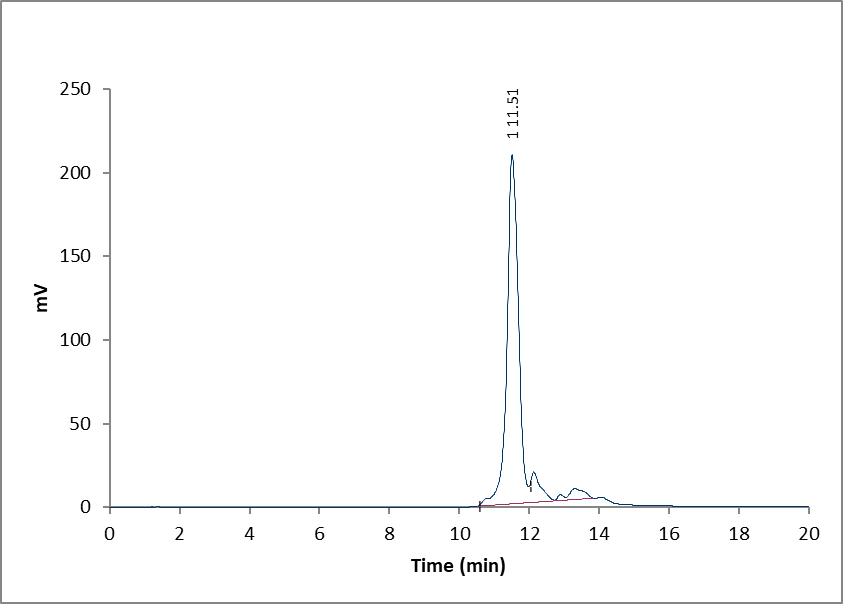
**

**B7**

**Fig. S4.** RP-HPLC chromatograms of **B1**–**B7**. Eluent A, 0.1% TFA in water; Eluent B, acetonitrile and monitoring at 340 nm with a gradient of 0%–80% eluent B over 20 min. Purities: **B1** (100%), **B2** (100%), **B3** (91.2%), **B4** (100%), **B5** (94.0%), **B6** (94.8%), and **B7** (100%).


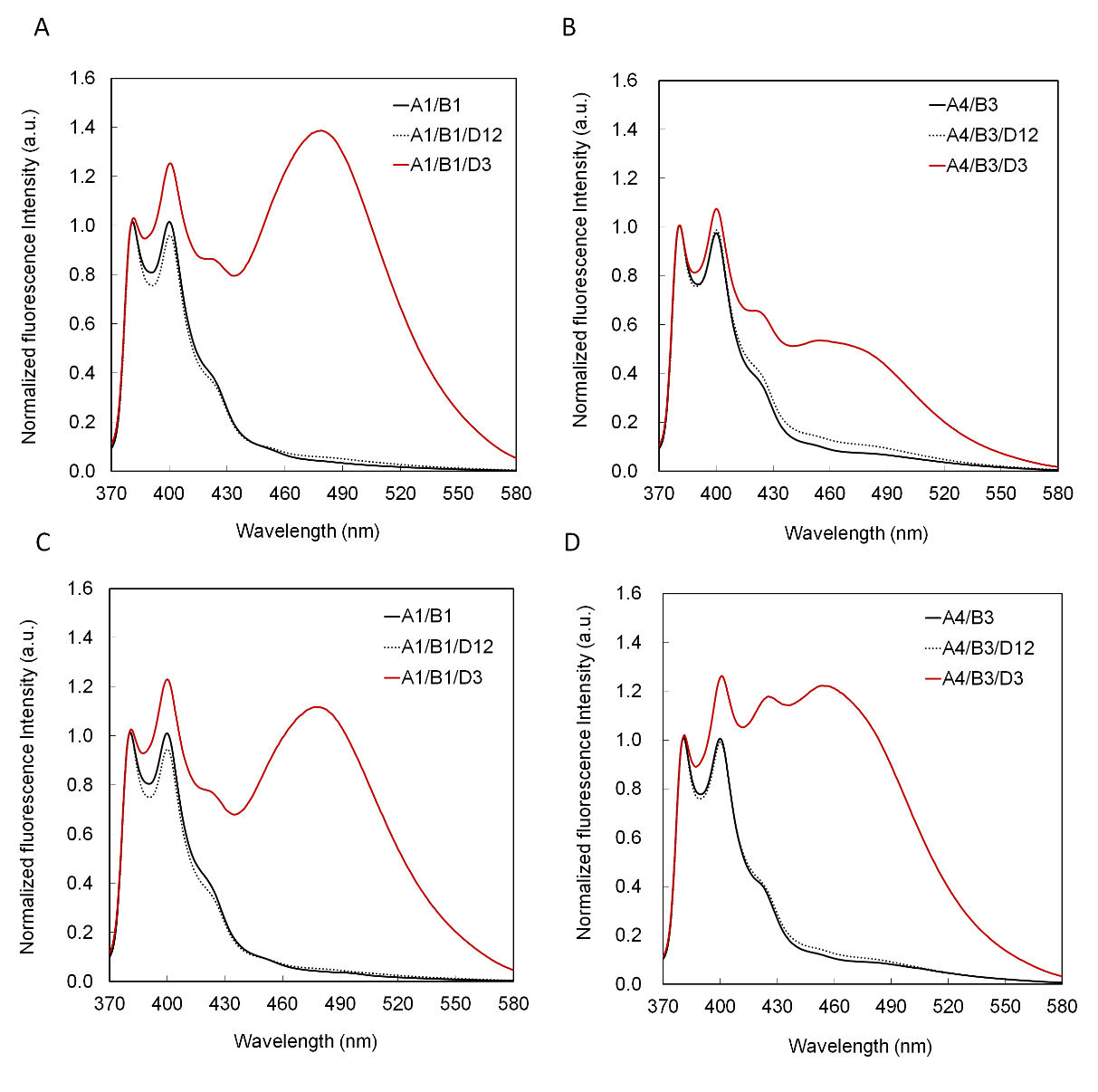


**Fig. S5.** Fluorescence spectra of equimolar mixtures of **A1**/**B1** (black solid line), **A1**/**B1**/**D3** (red solid line), and **A1**/**B1**/**D12** (black dotted line) after 20 min (A) and 24 h (C) in aqueous buffer solution. Fluorescence spectra of equimolar mixtures of **A4**/**B3** (black solid line), **A4**/**B3**/**D3** (red solid line), and **A4**/**B3**/**D12** (black dotted lines) after 20 min (B) and 24 h (D) in aqueous buffer solution.


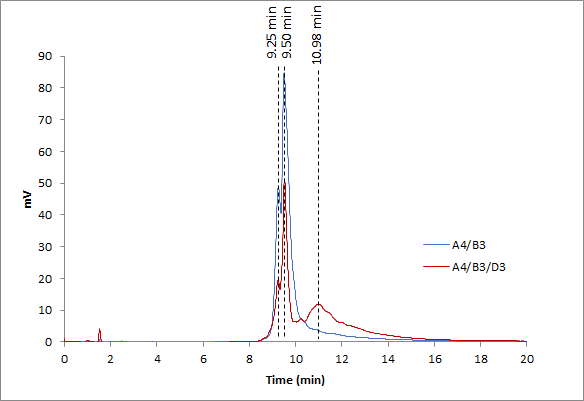


**Fig. S6.** RP-HPLC chromatograms of equimolar mixtures of **A4**/**B3** and **A4**/**B3**/**D3** after 12 h. Eluent A, 0.1% TFA in water; Eluent B, acetonitrile; monitoring at 340 nm with a gradient of 10%–80% eluent B over 20 min.


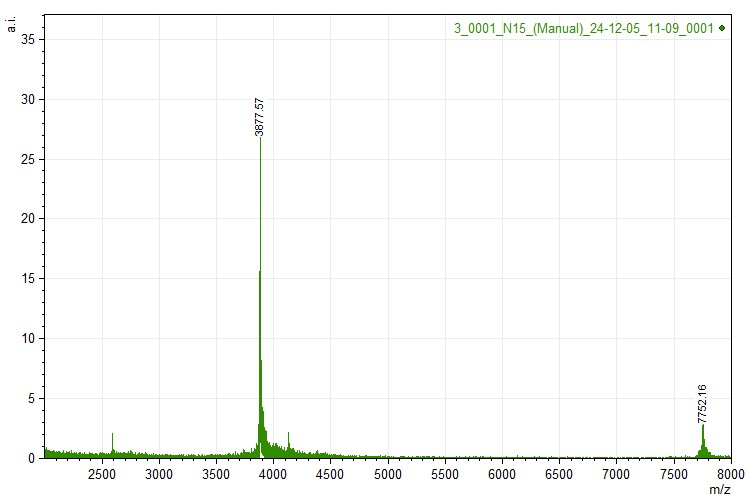


**Fig. S7.** MALDI-TOF mass spectrum of a fractionated sample of the peak at 10.98 min shown by HPLC of an equimolar mixture of **A4**/**B3**/**D3** after 24 h (Fig. S5). α-CHCA was used as a matrix. The peak at 3877.57 corresponds to a doubly charged peak at 7752.16.


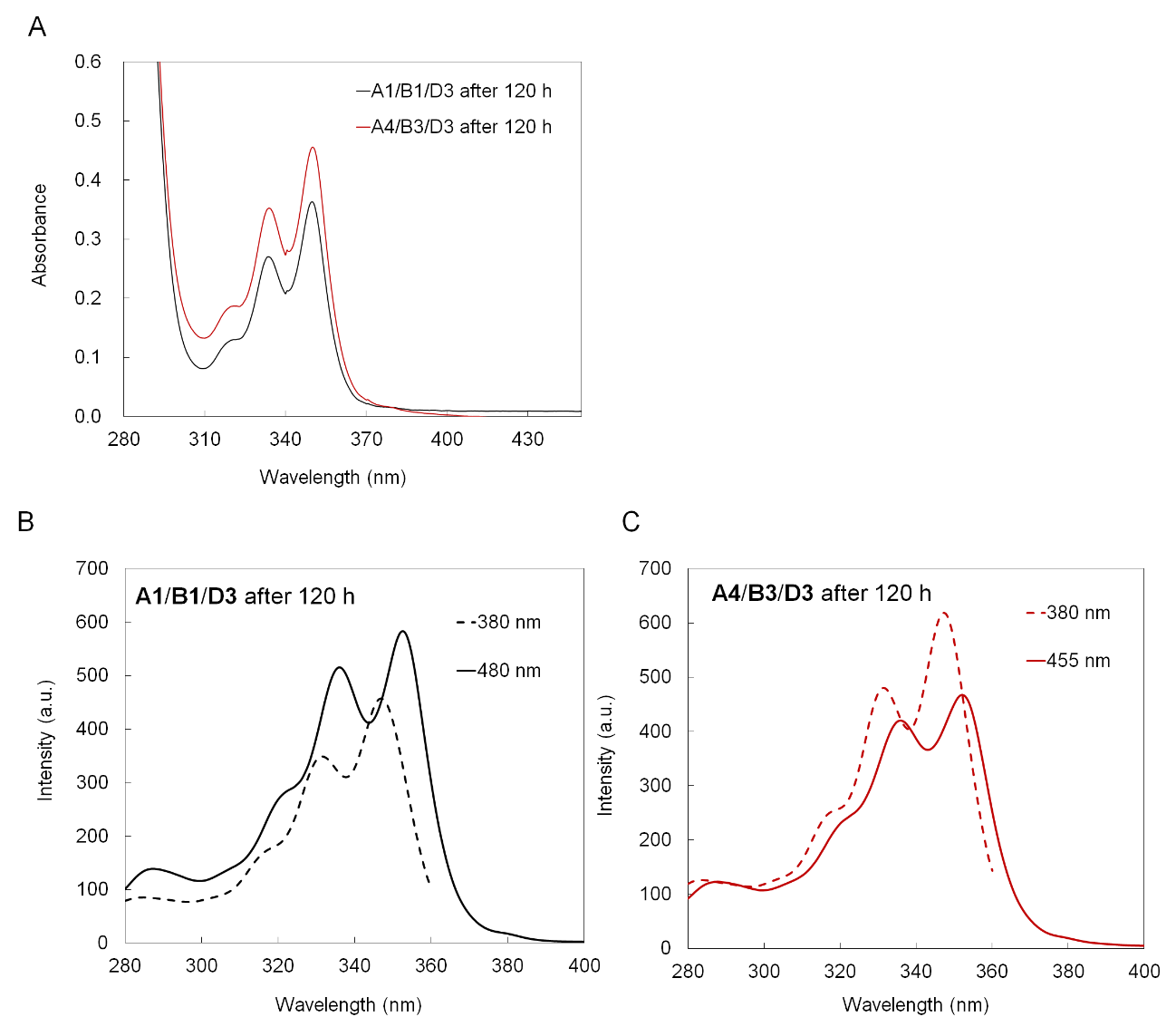


**Fig. S8.** (A) UV-vis spectra of the equimolar mixture of **A1**/**B1**/**D3** (black line) and the equimolar mixture of **A4**/**B3**/**D3** (red line) after 120 h. These measurements were conducted with a JASCO V-560 UV-vis spectrometer and a 1 cm quartz cell at wavelengths from 200 to 800 nm. Each of the final concentrations was 10 μM. The peaks derived from Pyr at 335 and 350 nm are confirmed. (B) Excitation spectra of **A1**/**B1**/**D3** and (C) **A4**/**B3**/**D3**. Emission wavelengths were 380 nm (dotted lines) and 480 nm (black solid line) or 455 nm (red solid line). These excitation spectra were measured at excitation wavelengths from 280 nm to 460 nm. The final concentration of each sample was 10 μM. All of these measurements were carried out in aqueous solution (10 mM PBS, pH 7.0) at 25 °C.


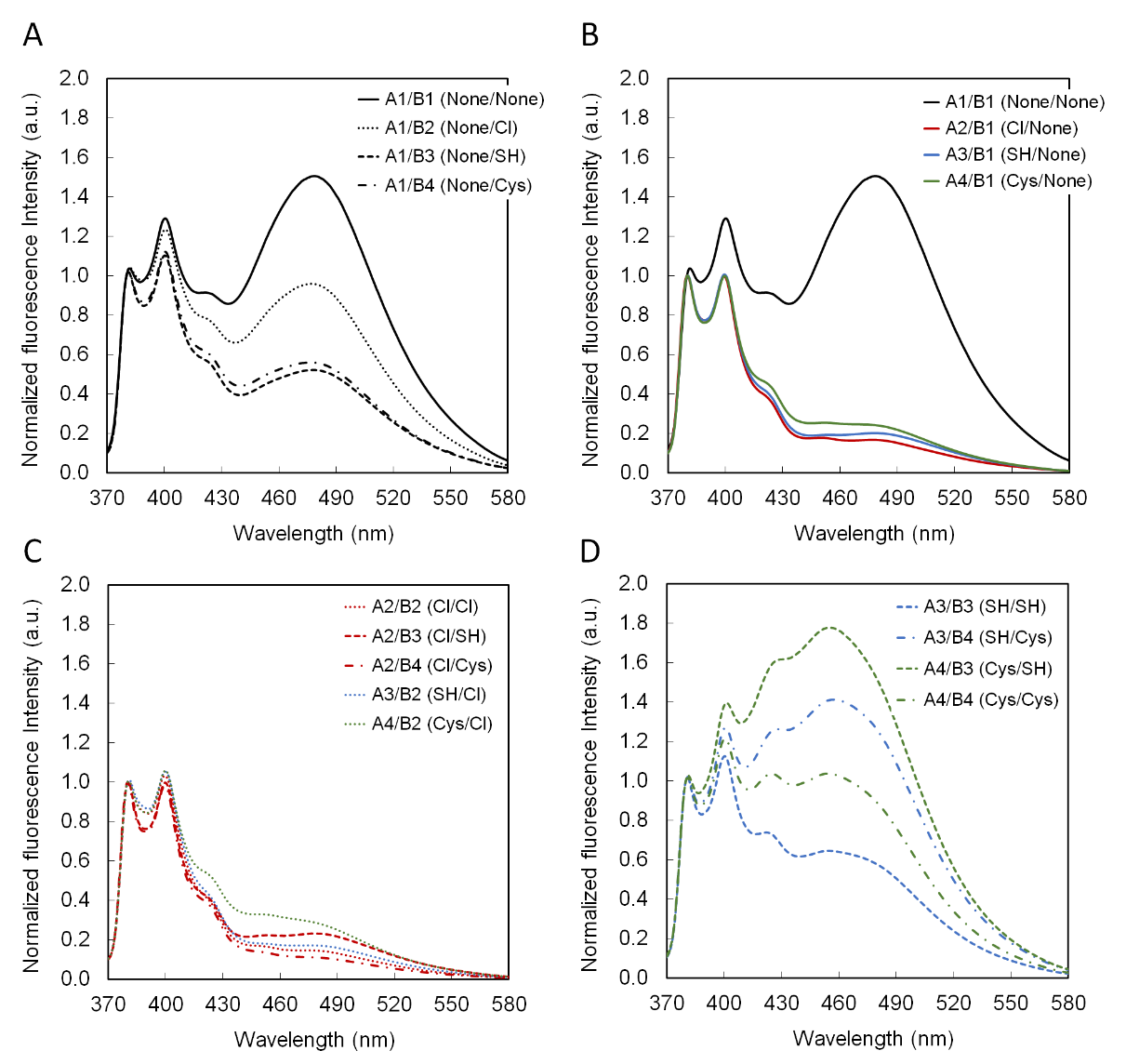


**Fig. S9.** (A–D) Fluorescence spectra of equimolar mixtures of **A1**–**A4**, **B1**–**B4**, and **D3** in aqueous buffer solution (10 mM PBS, pH 7.0). Fluorescence spectra were measured 120 h after mixing at 25°C. The final concentration of each of the **A** series, **B** series, and **D3** was 1.0 μM. Excitation wavelength was 350 nm.


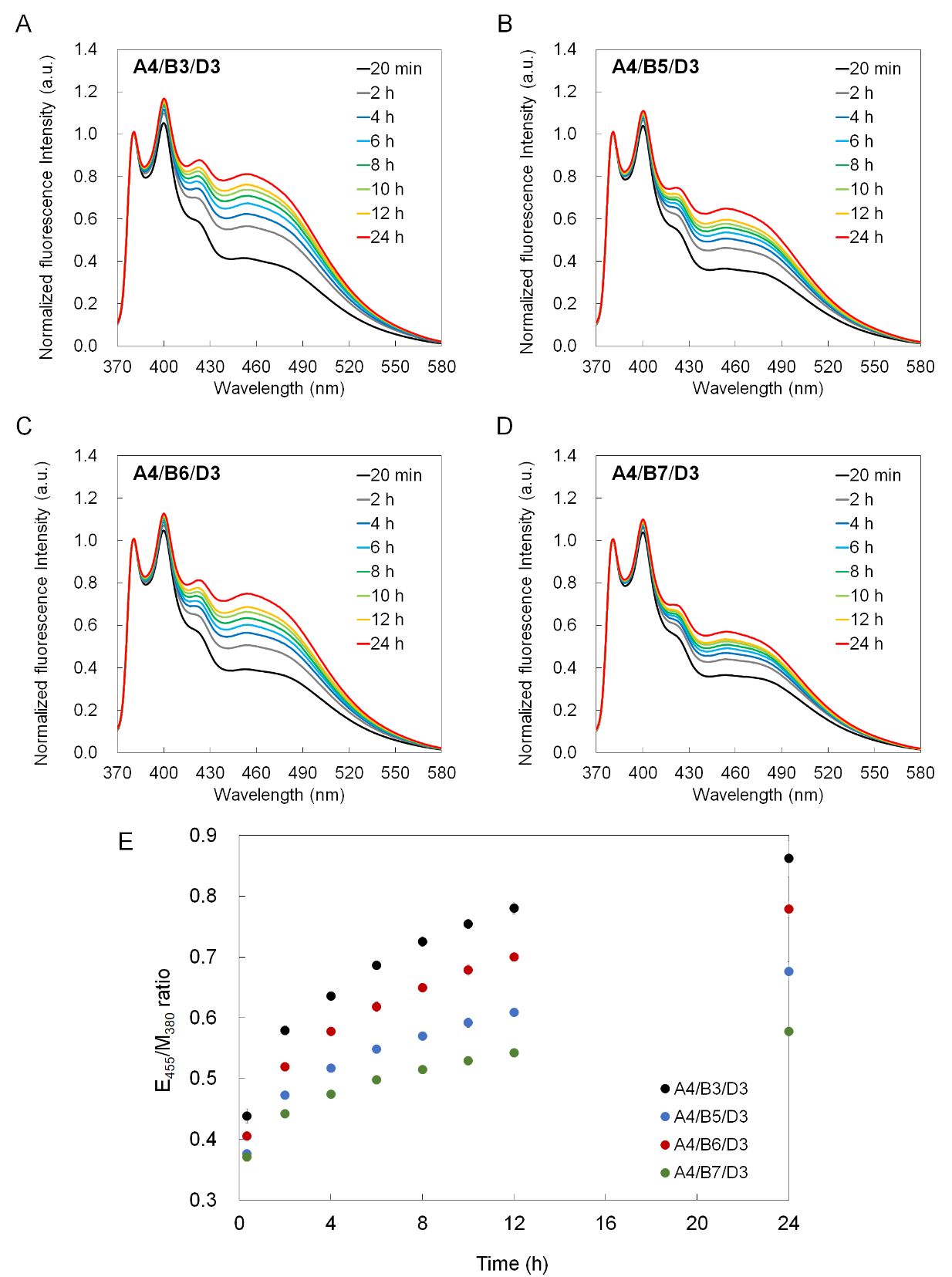


**Fig. S10.** Fluorescence spectra of equimolar mixtures of (A) **A4**/**B3**/**D3**, (B) **A4**/**B5**/**D3**, (C) **A4**/**B6**/**D3**, and (D) **A4**/**B7**/**D3** after various times. (E) Time course of E_455_/M_380_ ratio of equimolar mixtures of **A4**/**B3**/**D3**, **A4**/**B5**/**D3**, **A4**/**B6**/**D3**, and **A4**/**B7**/**D3** based on Fig. S10A**–**D**.**


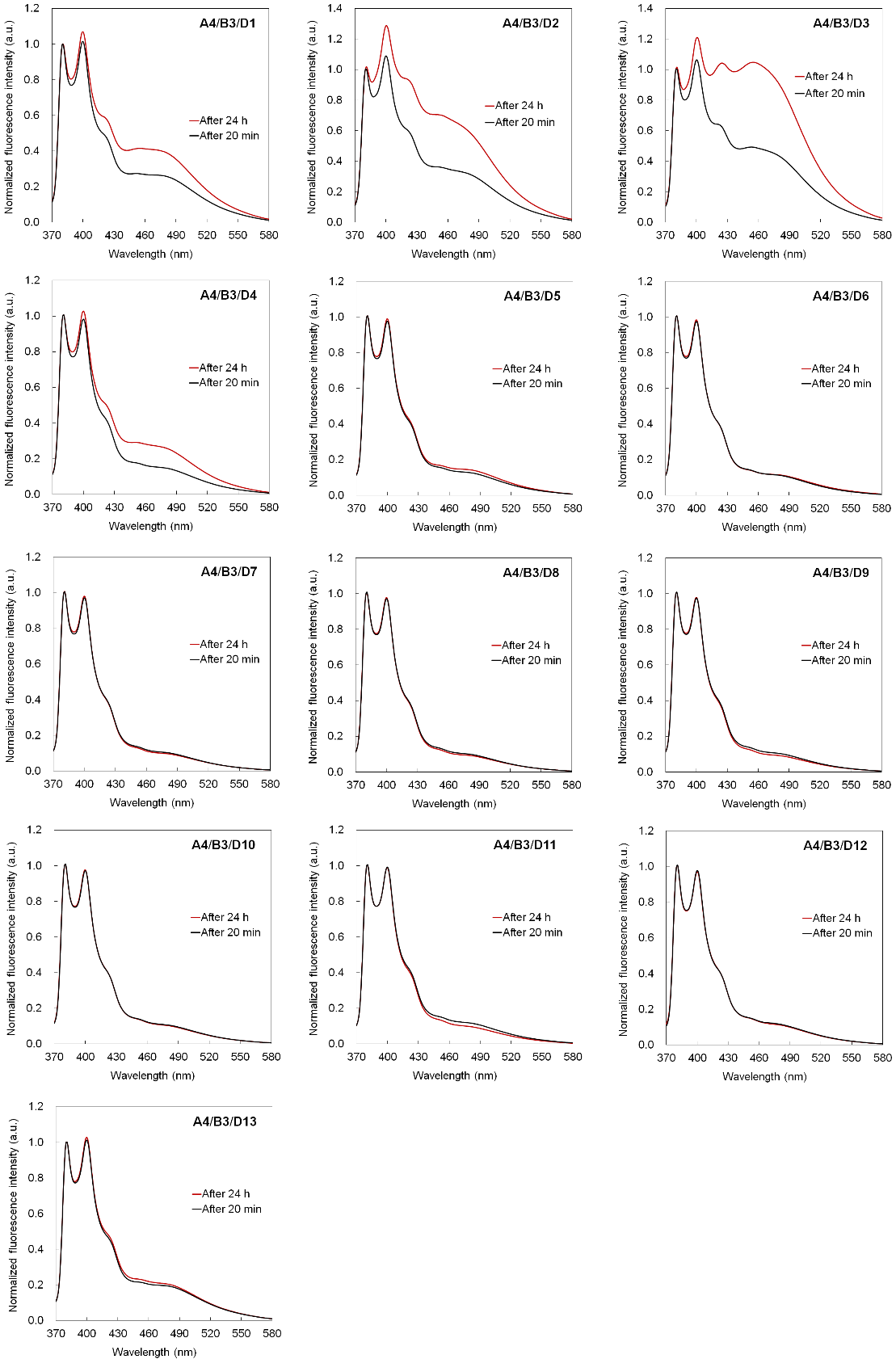


**Fig. S11.** Fluorescence spectra of equimolar mixtures of **A4**/**B3**/**D1–D13** after 20 min (black lines) and 24 h (red lines).


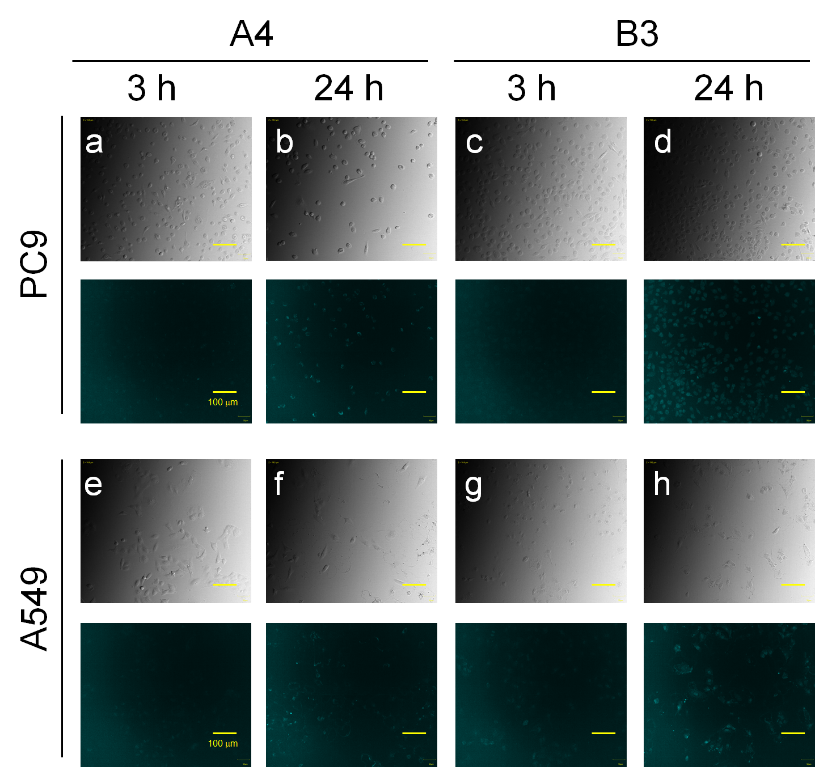


**Fig. S12.** Fluorescence images of live PC9 or A549 cells incubated with the 10 μM concentration of only **A4** or only **B3** probes. The images were acquired at 3 h and 24 h after probe addition. The scale bar represents 100 μm.


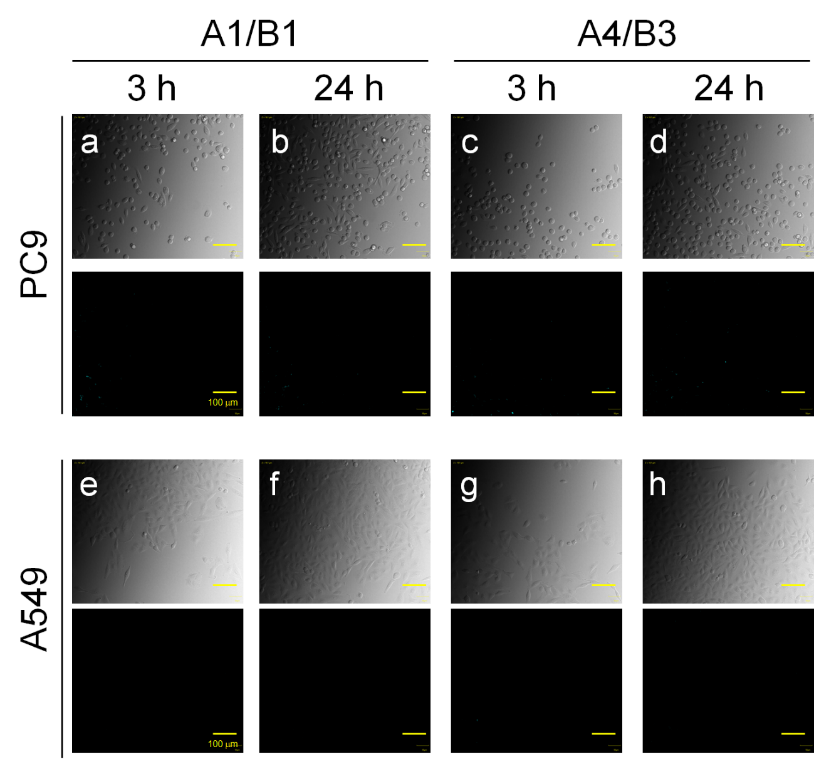


**Fig. S13.** Fluorescence images of live PC9 or A549 cells incubated with the 1 μM concentration of PNA twin probe. The images were acquired at 3 h and 24 h after probe addition. The scale bar represents 100 μm.


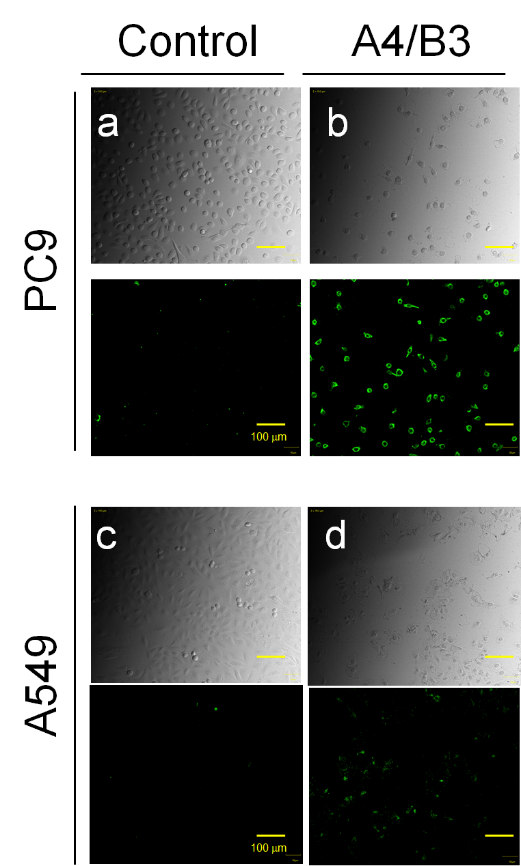


**Fig. S14.** Fluorescence images of apoptosis assay using FITC-Annexin V 24 h after treatment of 10 μM concentration of **A4**/**B3** twin probe. The scale bar represents 100 μm.
